# Geographic and age variations in mutational processes in colorectal cancer

**DOI:** 10.1038/s41586-025-09025-8

**Published:** 2025-04-23

**Authors:** Marcos Díaz-Gay, Wellington dos Santos, Sarah Moody, Mariya Kazachkova, Ammal Abbasi, Christopher D. Steele, Raviteja Vangara, Sergey Senkin, Jingwei Wang, Stephen Fitzgerald, Erik N. Bergstrom, Azhar Khandekar, Burçak Otlu, Behnoush Abedi-Ardekani, Ana Carolina de Carvalho, Thomas Cattiaux, Ricardo Cortez Cardoso Penha, Valérie Gaborieau, Priscilia Chopard, Christine Carreira, Saamin Cheema, Calli Latimer, Jon W. Teague, Anush Mukeriya, David Zaridze, Riley Cox, Monique Albert, Larry Phouthavongsy, Steven Gallinger, Reza Malekzadeh, Ahmadreza Niavarani, Marko Miladinov, Katarina Erić, Sasa Milosavljevic, Suleeporn Sangrajrang, Maria Paula Curado, Samuel Aguiar, Rui Manuel Reis, Monise Tadin Reis, Luis Gustavo Romagnolo, Denise Peixoto Guimarães, Ivana Holcatova, Jaroslav Kalvach, Carlos Alberto Vaccaro, Tamara Alejandra Piñero, Beata Świątkowska, Jolanta Lissowska, Katarzyna Roszkowska-Purska, Antonio Huertas-Salgado, Tatsuhiro Shibata, Satoshi Shiba, Surasak Sangkhathat, Taned Chitapanarux, Gholamreza Roshandel, Patricia Ashton-Prolla, Daniel C. Damin, Francine Hehn de Oliveira, Laura Humphreys, Trevor D. Lawley, Sandra Perdomo, Michael R. Stratton, Paul Brennan, Ludmil B. Alexandrov

**Affiliations:** 1 Department of Cellular and Molecular Medicine, University of California San Diego, La Jolla, CA USA; 2 Department of Bioengineering, University of California San Diego, La Jolla, CA USA; 3 Moores Cancer Center, University of California San Diego, La Jolla, CA USA; 4 Digital Genomics Group, Structural Biology Program, Spanish National Cancer Research Center (CNIO), Madrid, Spain; 5 Genomic Epidemiology Branch, International Agency for Research on Cancer (IARC/WHO), Lyon, France; 6 Cancer, Ageing and Somatic Mutation, Wellcome Sanger Institute, Cambridge, UK; 7 Biomedical Sciences Graduate Program, University of California San Diego, La Jolla, CA USA; 8 Division of Cancer Epidemiology and Genetics, National Cancer Institute, Bethesda, MD USA; 9 Department of Health Informatics, Graduate School of Informatics, Middle East Technical University, Ankara, Turkey; 10 Evidence Synthesis and Classification Branch, International Agency for Research on Cancer (IARC/WHO), Lyon, France; 11 Clinical Epidemiology, N. N. Blokhin National Medical Research Centre of Oncology, Moscow, Russia; 12 Ontario Tumour Bank, Ontario Institute for Cancer Research, Toronto, Ontario Canada; 13 Centre for Biodiversity Genomics, University of Guelph, Guelph, Ontario Canada; 14 Lunenfeld-Tanenbaum Research Institute, Sinai Health System, Toronto, Ontario Canada; 15 Digestive Oncology Research Center, Digestive Disease Research Institute, Tehran University of Medical Sciences, Tehran, Iran; 16 Clinic for Digestive Surgery—First Surgical Clinic, University Clinical Centre of Serbia, Belgrade, Serbia; 17 Department of Pathology, University Clinical Centre of Serbia, Belgrade, Serbia; 18 International Organization for Cancer Prevention and Research, Belgrade, Serbia; 19 National Cancer Institute, Bangkok, Thailand; 20 Department of Epidemiology, A. C. Camargo Cancer Center, Sao Paulo, Brazil; 21 Colon Cancer Reference Center, A. C. Camargo Cancer Center, Sao Paulo, Brazil; 22 Molecular Oncology Research Center, Barretos Cancer Hospital, Barretos, Brazil; 23 Life and Health Sciences Research Institute (ICVS), School of Medicine, Minho University, Braga, Portugal; 24 Department of Pathology, Barretos Cancer Hospital, Barretos, Brazil; 25 Department of Colorectal Oncology Surgery, Barretos Cancer Hospital, Barretos, Brazil; 26 Department of Endoscopy, Barretos Cancer Hospital, Barretos, Brazil; 27 Department of Oncology, 2nd Faculty of Medicine, Charles University and University Hospital Motol, Prague, Czech Republic; 28 Institute of Hygiene and Epidemiology, 1st Faculty of Medicine, Charles University, Prague, Czech Republic; 29 Surgery Department, 2nd Faculty of Medicine, Charles University and Central Military Hospital, Prague, Czech Republic; 30 2nd Faculty of Medicine, Charles University and Motol University Hospital, Prague, Czech Republic; 31 Institute of Animal Physiology and Genetics Czech Academy of Science, Libechov, Czech Republic; 32 Clinical Center ISCARE, Prague, Czech Republic; 33 Instituto de Medicina Traslacional e Ingeniería Biomédica (IMTIB)–CONICET–Universidad Hospital Italiano de Buenos Aires (UHIBA) y Hospital Italiano de Buenos Aires (HIBA), Buenos Aires, Argentina; 34 Department of Environmental Epidemiology, Nofer Institute of Occupational Medicine, Łódź, Poland; 35 The Maria Sklodowska-Cure National Research Institute of Oncology, Warsaw, Poland; 36 Department of Pathology, The Maria Sklodowska-Cure National Research Institute of Oncology, Warsaw, Poland; 37 Oncological Pathology Group, Terry Fox National Tumor Bank (Banco Nacional de Tumores Terry Fox), National Cancer Institute, Bogotá, Colombia; 38 Laboratory of Molecular Medicine, The Institute of Medical Science, The University of Tokyo, Minato-ku, Japan; 39 Division of Cancer Genomics, National Cancer Center Research Institute, Chuo-ku, Japan; 40 Translational Medicine Research Center, Faculty of Medicine, Prince of Songkla University, Hat Yai, Thailand; 41 Department of Biomedical Sciences and Biomedical Engineering, Faculty of Medicine, Prince of Songkla University, Hat Yai, Thailand; 42 Department of Surgery, Faculty of Medicine, Prince of Songkla University, Hat Yai, Thailand; 43 Department of Internal Medicine, Faculty of Medicine, Chiang Mai University, Chiang Mai, Thailand; 44 Golestan Research Center of Gastroenterology and Hepatology, Golestan University of Medical Sciences, Gorgan, Iran; 45 Department of Genetics, Universidade Federal do Rio Grande do Sul (UFRGS), Porto Alegre, Brazil; 46 Medical Genetics Service, Hospital de Clínicas de Porto Alegre (HCPA), Porto Alegre, Brazil; 47 Department of Surgery, Division of Colorectal Surgery, Hospital de Clínicas de Porto Alegre (HCPA), Porto Alegre, Brazil; 48 Department of Pathology, Anatomic Pathology, Hospital de Clínicas de Porto Alegre (HCPA), Porto Alegre, Brazil; 49 Parasites and Microbes, Wellcome Sanger Institute, Cambridge, UK; 50 Sanford Stem Cell Institute, University of California San Diego, La Jolla, CA USA

**Keywords:** Colorectal cancer, Cancer genomics, Cancer epidemiology

## Abstract

Incidence rates of colorectal cancer vary geographically and have changed over time^1^. Notably, in the past two decades, the incidence of early-onset colorectal cancer, which affects individuals below 50 years of age, has doubled in many countries^2–5^. The reasons for this increase are unknown. Here we investigate whether mutational processes contribute to geographic and age-related differences by examining 981 colorectal cancer genomes from 11 countries. No major differences were found in microsatellite-unstable cancers, but variations in mutation burden and signatures were observed in the 802 microsatellite-stable cases. Multiple signatures, most with unknown aetiologies, exhibited varying prevalence in Argentina, Brazil, Colombia, Russia and Thailand, indicating geographically diverse levels of mutagenic exposure. Signatures SBS88 and ID18, caused by the bacteria-produced mutagen colibactin^6,7^, had higher mutation loads in countries with higher colorectal cancer incidence rates. SBS88 and ID18 were also enriched in early-onset colorectal cancers, being 3.3 times more common in individuals who were diagnosed before 40 years of age than in those over 70 years of age, and were imprinted early during colorectal cancer development. Colibactin exposure was further linked to *APC* driver mutations, with ID18 being responsible for about 25% of *APC* driver indels in colibactin-positive cases. This study reveals geographic and age-related variations in colorectal cancer mutational processes, and suggests that mutagenic exposure to colibactin-producing bacteria in early life may contribute to the increasing incidence of early-onset colorectal cancer.

## Abstract (web summary)

Analysis of genomic data from 981 colorectal cancers from participants in 11 countries reveals variations in mutational signatures of microsatellite-stable cancers that are dependent on geographical origin and age at which the cancer was diagnosed.

## Main

The age-standardized incidence rates (ASRs) for most adult cancers vary across different geographic locations and can change over time^1^. Despite extensive epidemiological research, the underlying causes for many of these variations remain unclear. However, they are thought to be due to exogenous environmental or lifestyle carcinogenic exposures, which are, in principle, preventable^8^. Many well-known exogenous carcinogens are also mutagens^9,10^ that can imprint characteristic patterns of somatic mutations—mutational signatures—in the genome. Therefore, a complementary approach to conventional epidemiology for investigating unknown causes of cancer is the characterization of mutational signatures in the genomes of cancer and normal cells^11–13^. The Mutographs Cancer Grand Challenge project^14^ has implemented this strategy of ‘mutational epidemiology’ by sequencing cancers from geographic areas of differing incidence rates, using mutational signature analysis to reveal the mutational processes that have been operative, with results so far from cancers of the oesophagus^11^, kidney^13^ and head and neck^15^.

Colorectal cancer incidence rates differ markedly by geographic location and have changed substantially in some countries over the past 70 years^5^. For instance, the ASRs for colorectal cancer in North America and in most European countries peaked in the 1980s and 1990s and have been declining since, whereas countries in East Asia such as Japan and South Korea have been steadily increasing over the past seven decades^1^. Moreover, in the past 20 years, there has been a notable global increase in the incidence of early-onset colorectal cancer^4,5^, typically defined as colorectal cancer in adults under 50 years of age. This was first reported in the USA^2^ and subsequently observed in Australia, Canada, Japan and multiple European countries^3,4^. Although epidemiological studies have identified multiple risk factors for colorectal cancer, specific risk factors for early-onset colorectal cancer remain largely unidentified, with the exception of family history and hereditary predisposition. The latter is predominantly attributable to Lynch syndrome, which is characterized by cancers of the proximal colon that are deficient in DNA mismatch repair^16,17^ and is therefore unlikely to be implicated in the recent increase in early-onset colorectal cancer, which is mainly enriched in sporadic, DNA mismatch repair-proficient cancers that affect the distal colon and rectum^18,19^.

Previous colorectal cancer whole-genome sequencing studies have largely focused on cases from North America and Europe, including the USA^20,21^, UK^22,23^, Netherlands^24–27^ and Sweden^28^, and incorporated limited numbers of early-onset cases^21–23,27,28^. Here we examine colorectal cancer genomes from 11 countries on 4 continents to investigate whether variation in mutational processes contributes to geographic and age-related differences in incidence rates.

## Study design

**Fig. 1:**
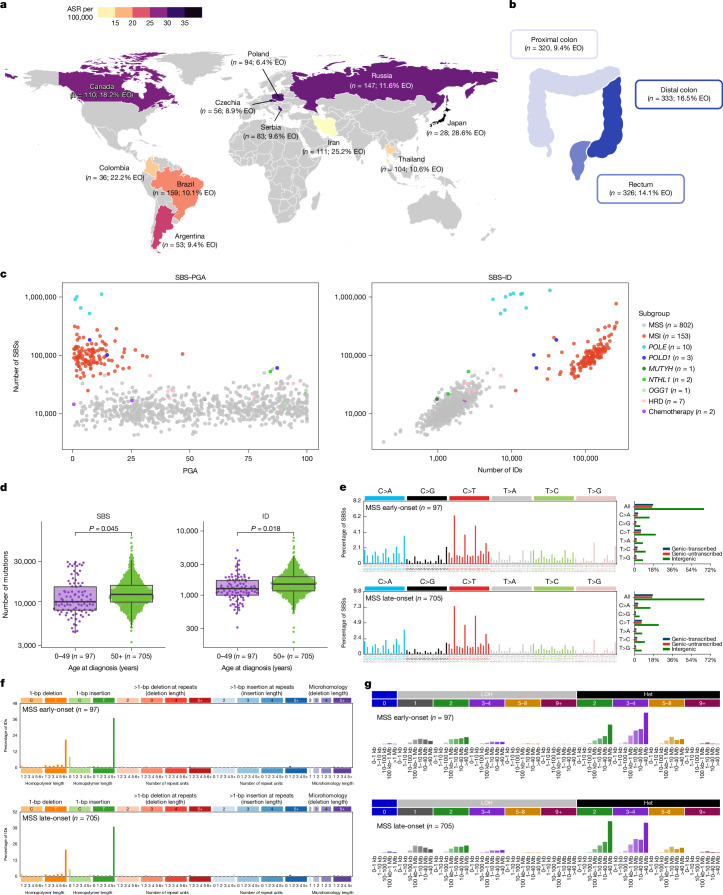
Geographic, clinical and molecular characterization of the Mutographs colorectal cancer cohort. **a**, Geographic distribution of the 981 patients with primary colorectal cancer across 4 continents and 11 countries, indicating the total number of cases and the percentage of early-onset cases (EO; onset before 50 years of age). Countries were coloured according to their ASR per 100,000 individuals. **b**, Tumour subsite distribution of the cohort across the colorectum (two cases had unspecified subsites). Subsites were coloured according to the percentage of early-onset cases. **c**, Distribution of molecular subgroups according to the total number of SBSs, IDs and percentage of genome aberrated (PGA). Cases for which tumour purity was insufficient to determine an accurate copy number profile or without large CNs (65 out of 981 cases) were excluded from the SBS–PGA panel. **d**, Distribution of SBS and ID across early-onset (less than 50 years of age; purple) and late-onset (aged 50 years or older; green) MSS colorectal tumours. Statistically significant differences were evaluated using multivariable linear regression models adjusted by sex, country, tumour subsite and tumour purity. In box plots, the horizontal line indicates the median, the upper and lower ends of the box indicate the 25th and 75th percentiles. Whiskers show 1.5 × the interquartile range, and values outside the whiskers are shown as individual data points. **e**–**g**, Average mutational profiles of early- and late-onset MSS tumours for SBSs (SBS-288 mutational context (**e**)), IDs (ID-83 (**f**)) and CNs (CN-68 (**g**)). Het, heterozygous; LOH, loss of heterozygosity.

In total, 981 colorectal cancers (45.7% female) were collected from intermediate-incidence countries with ASRs of 13 to 20 per 100,000 people (Iran, Thailand, Colombia and Brazil) and high-incidence countries with ASRs greater than 24 (Argentina, Canada, Russia, Serbia, Czech Republic, Poland and Japan), including the highest ASR of 37 in Japan^1^ (Fig. 1a and Supplementary Table 1). Out of the 981 cases, 320 were from the proximal colon, 333 were from the distal colon, 326 were from the rectum, and 2 were from unspecified subsites (Fig. 1b). There were 132 early-onset cases, which were 1.88-fold enriched in the distal colon and rectum compared with the proximal colon (*P* = 0.006). All cancers and their matched normal samples underwent whole-genome sequencing, achieving a median coverage of 53-fold and 27-fold, respectively.

## Molecular classification

**Extended Data Fig. 1:**
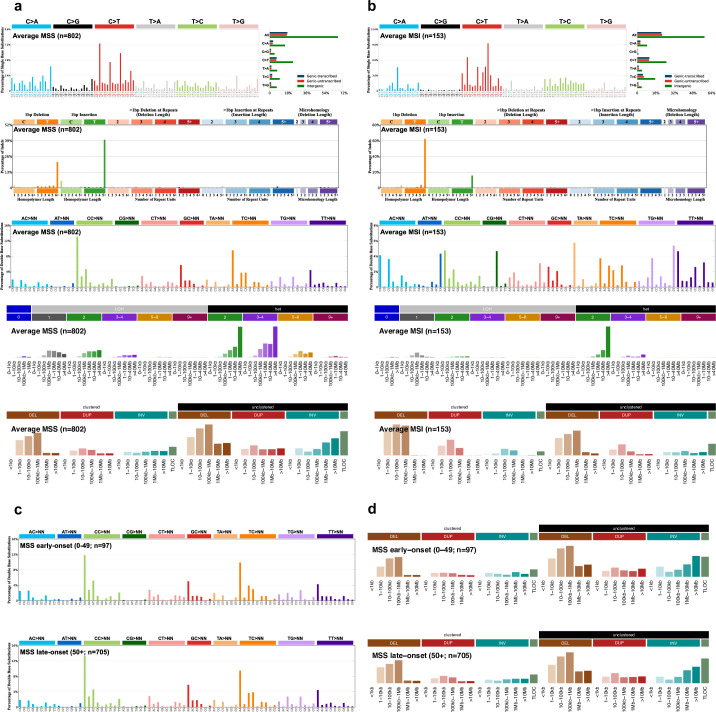
Mutational profiles across molecular subtypes and ages of onset. **a-b**, Average mutational profiles of microsatellite stable (MSS; **a**) and microsatellite unstable (MSI; **b**) colorectal tumors for single base substitutions (SBS-288 mutational context), small insertions and deletions (ID-83 mutational context), doublet base substitutions (DBS-78 mutational context), copy number alterations (CN-68 mutational context), and structural variants (SV-38 mutational context). **c-d**, Average mutational profiles of early-onset and late-onset MSS colorectal tumors for doublet base substitutions (**c**) and structural variants (**d**).

The 981 colorectal cancers were divided into known molecular subtypes on the basis of their somatic mutation burdens and profiles. Consistent with prior studies^20,29^, two main subtypes were identified: DNA mismatch repair-proficient cancers, also known as microsatellite stable (MSS), and DNA mismatch repair-deficient cancers, often referred to as tumours showing microsatellite instability (MSI). MSS samples (*n* = 802, 81.8%; Fig. 1c) were characterized by a lower burden of single base substitutions (SBSs; median: 12,054) and small insertions and deletions (IDs, also known as indels; median: 1,451), and a higher burden of large-scale genomic aberrations (median: 53.5% of genome altered). By contrast, MSI samples (*n* = 153, 15.6%) exhibited higher SBS and ID burdens (median: 95,426 and 125,100, respectively) with limited genomic aberrations (median: 7.0%). As expected, the average mutational profiles of MSS and MSI colorectal tumours were different (Extended Data Fig. 1a,b).

MSI samples were found predominantly in the proximal colon (odds ratio (OR) = 12.2, *P* = 3.8 × 10^−27^) and were more common in early-onset cases (OR = 2.6, *P* = 0.001). Notably, 31 out of 153 MSI cases (20.3%), including 13 out of 28 MSI early-onset cases (46.4%), carried germline pathogenic variants in DNA mismatch repair genes consistent with Lynch syndrome (Supplementary Table 2). After excluding all cases attributed to Lynch syndrome, there was no enrichment of MSI cancers in early-onset cases (*P* > 0.05). Deficiencies of other DNA repair mechanisms were observed in 24 out of the 981 cancers (2.4%), including ultra-hypermutated cases with mutations in *POLE* (*n* = 10, 1.0%) and *POLD1* (*n* = 3, 0.3%) polymerase genes, homologous recombination-deficient (HRD) cases (*n* = 7, 0.7%), and cases with mutations in the base excision repair genes *MUTYH* (*n* = 1, 0.1%), *NTHL1* (*n* = 2, 0.2%) and *OGG1* (*n* = 1, 0.1%) (Supplementary Tables 3 and 4, Supplementary Figs. 1–3 and Methods).

The mutational catalogues of DNA repair-deficient cancers are dominated by somatic mutations resulting from the failed repair process, rendering it difficult to characterize mutational processes that are unrelated to this failure^30^. To enable investigation of the latter, we therefore focused the main analyses on DNA repair-proficient colorectal cancers, while reporting DNA repair-deficient cases in the Supplementary Note. Two cases treated with chemotherapy for prior cancers were also excluded, as their mutation profiles were dominated by the mutational signatures of chemotherapy agents^21,31^ (Supplementary Fig. 4). The remaining cohort consisted of 802 treatment-naive DNA repair-proficient colorectal cancers, including 97 early-onset cases.

**Extended Data Fig. 2:**
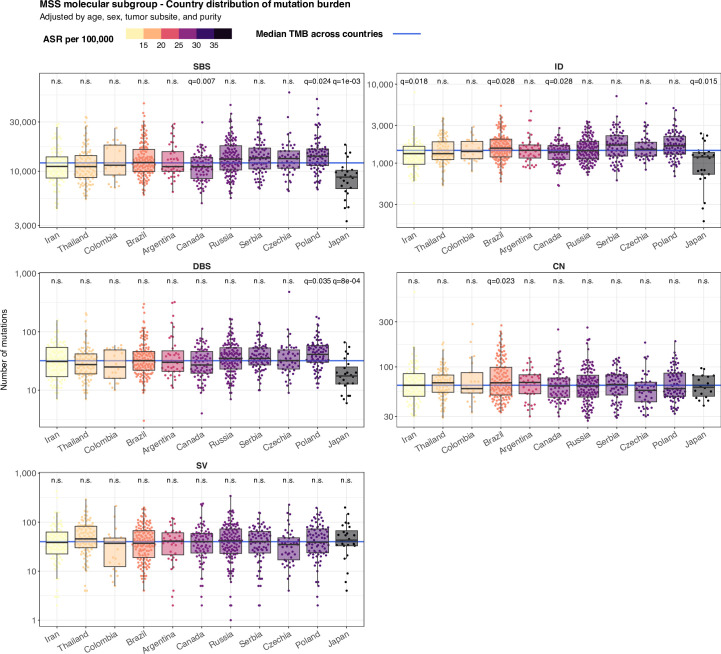
Geographic distribution of mutation burden. Box plots indicating the distribution of single base substitutions (SBS), small insertions and deletions (ID), doublet base substitutions (DBS), copy number alterations (CN), and structural variants (SV) across countries for microsatellite stable (MSS) colorectal tumors. Box plots and data points representing total number of mutations for each variant type were colored according to each country’s colorectal cancer age-standardized incidence rates (ASR) per 100,000 individuals. A horizontal blue line indicates the median mutation burden for each variant type. Statistically significant differences were evaluated using multivariable linear regression models comparing each country to all others and adjusted by age of diagnosis, sex, tumor subsite, and tumor purity. P-values were adjusted for multiple comparisons using the Benjamini-Hochberg method based on the total number of countries assessed and reported as q-values. The line within the box indicates the median, while the upper and lower ends indicate the 25^th^ and 75^th^ percentiles. Whiskers show 1.5 × interquartile range, and values outside are shown as individual data points.

**Extended Data Fig. 3:**
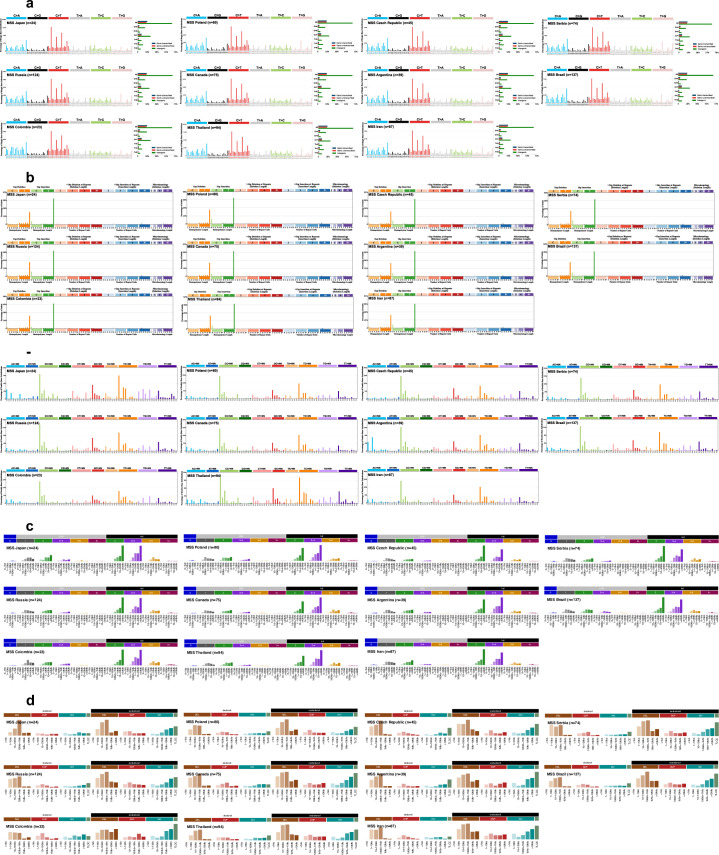
Geographic distribution of mutational profiles. **a-e**, Average mutational profiles of microsatellite stable (MSS) colorectal tumors for single base substitutions (SBS-288 mutational context; **a**), small insertions and deletions (ID-83 mutational context; **b**), doublet base substitutions (DBS-78 mutational context; **c**), copy number alterations (CN-68 mutational context; **d**), and structural variants (SV-38 mutational context; **e**).

After adjustment for sex, country, tumour subsite and tumour purity (Methods), early-onset cancers showed reduced burdens of SBSs (fold change (FC) = 0.92, *P* = 0.045) and IDs (FC = 0.90, *P* = 0.018; Fig. 1d) but not of doublet base substitutions (DBSs), copy number alterations (CNs) or structural variants (SVs) when compared with late-onset cases (*P* > 0.05). Nevertheless, the average mutation spectra of early-onset and late-onset cancers were remarkably similar for all types of somatic mutations (cosine similarity > 0.97; Fig. 1e–g and Extended Data Fig. 1c,d). Mutation burden also varied substantially for specific countries when compared to all others, including Canada (lower SBS and ID burdens), Poland (higher SBS and DBS), Japan (lower SBS, ID, DBS), Iran (lower ID) and Brazil (higher ID and CN; Extended Data Fig. 2). However, mutation profiles were generally consistent across all countries (Extended Data Fig. 3).

## Repertoire of mutational signatures

**Extended Data Fig. 4:**
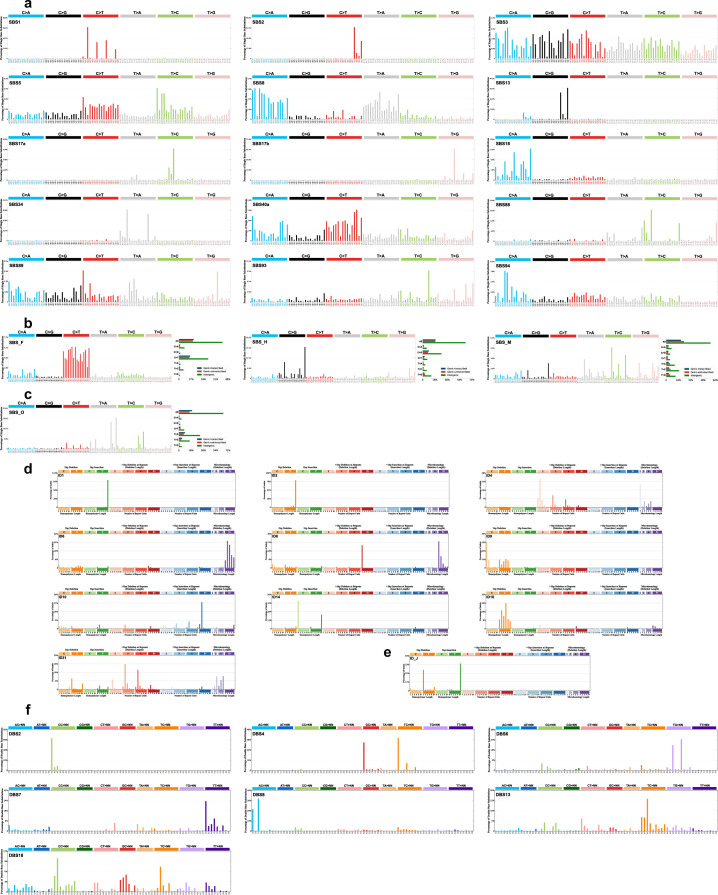
Mutational signatures of small mutational events identified in microsatellite stable colorectal cancers. **a-c**, Mutational profiles of single base substitution (SBS) signatures, including COSMICv3.4 reference signatures (**a**), previously reported signatures not present in COSMIC (**b**), and novel signature SBS_O (**c**). **d-e**, Mutational profiles of small insertions and deletions (ID) signatures, including COSMICv3.4 signatures (**d**) and novel signature ID_J (**e**). **f**, Mutational profiles of doublet base substitution (DBS) signatures, all previously reported in COSMIC.

**Extended Data Fig. 5:**
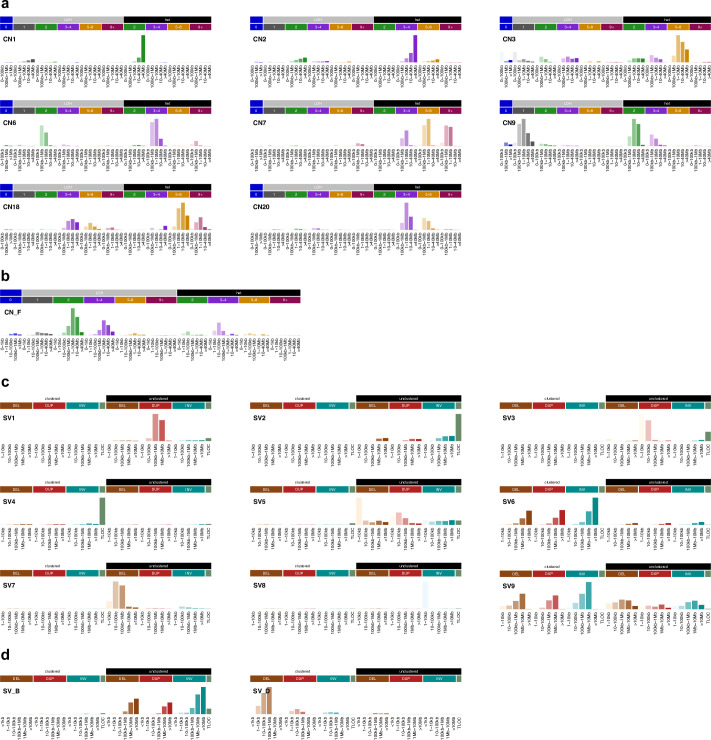
Mutational signatures of large mutational events identified in microsatellite stable colorectal cancers. **a-b**, Mutational profiles of copy number (CN) signatures, including COSMICv3.4 reference signatures (**a**) and novel signature CN_F (**b**). **c-d**, Mutational profiles of structural variant (SV) signatures, including COSMIC signatures (**c**) and novel signatures SV_B and SV_D (**d**).

A total of 16 SBS, 10 ID, 4 DBS, 6 CN and 6 SV de novo mutational signatures were extracted from the 802 MSS colorectal cancers and subsequently decomposed into a combination of previously reported reference signatures and potential novel signatures (Supplementary Tables 5–15 and Methods). The 16 de novo SBS signatures encompassed 15 COSMICv3.4 signatures (Extended Data Fig. 4a and Supplementary Table 10), including those previously associated with clock-like mutational processes (SBS1 and SBS5)^32^, APOBEC deamination (SBS2 and SBS13)^32^, deficient homologous recombination (SBS3)^32^, reactive oxygen species (SBS18)^33^, exposure to the mutagenic agent colibactin synthesized by *Escherichia coli* and other microorganisms carrying an approximately 40-kb polyketide synthase (*pks*) pathogenicity island (SBS88)^6,7^, and mutational processes of unknown causes (SBS8, SBS17a, SBS17b, SBS34, SBS40a, SBS89, SBS93 and SBS94)^6,13,21,33,34^. Three previously described signatures of unknown origin^22^ (SBS_F, SBS_H and SBS_M; Extended Data Fig. 4b) and a novel signature (SBS_O; Extended Data Fig. 4c) were also detected. SBS_O corresponds to a refined version of a previously reported signature of unknown aetiology^21^ (SBS41; Methods). With respect to IDs, DBSs, CNs and SVs, most de novo extracted mutational signatures were highly similar to, or directly reconstructed by, COSMICv3.4 reference signatures (Extended Data Figs. 4d–f and 5 and Supplementary Table 10), with the exception of an ID signature (ID_J) characterized by deletions of isolated Ts and insertions of Ts in long repetitive regions resembling a previously reported signature^6^ (Extended Data Fig. 4e), and three novel signatures from large mutational events (CN_F, SV_B, SV_D; Extended Data Fig. 5b,d), which were extracted owing to the extended contexts used in our signature analysis (Methods).

## Geographic variation of signatures

**Fig. 2:**
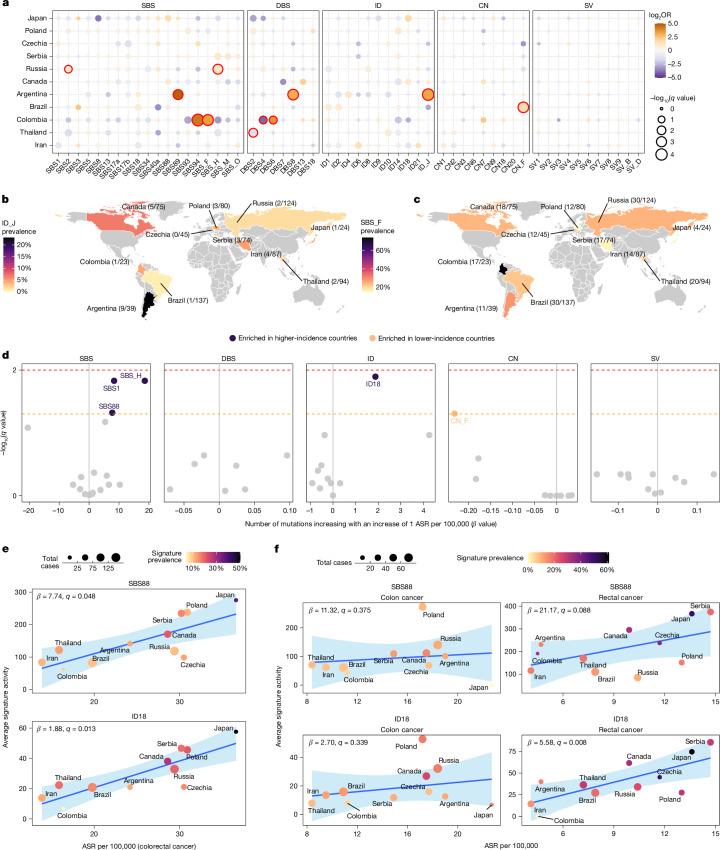
Geographic variation of mutational signatures in MSS colorectal cancers. **a**, Variation of signature prevalence in specific countries compared to all others. Statistically significant enrichments were evaluated using multivariable logistic regression models adjusted by age of diagnosis, sex, tumour subsite and tumour purity. Firth’s bias-reduced logistic regressions were used for regression presenting complete or quasi-complete separation. Data points were coloured according to the odds ratio (OR), with size representing statistical significance. *P* values were adjusted for multiple comparisons using the Benjamini–Hochberg method based on the total number of signatures considered per variant type and the total number of countries assessed, and reported as *q* values. *q* values <0.05 were considered statistically significant and marked in red. **b**,**c**, Geographic distribution of the ID_J (**b**) and SBS_F (**c**) mutational signatures. Countries were coloured on the basis of signature prevalence. **d**, Association of signature activities with ASR. Statistically significant associations were evaluated using multivariable linear regression models adjusted by age of diagnosis, sex, tumour subsite and tumour purity. *P* values were adjusted for multiple comparisons using the Benjamini–Hochberg method based on the total number of signatures considered per variant type and reported as *q* values. Dashed lines indicate *q* values of 0.05 (orange) and 0.01 (red). **e**,**f**, Association of the mutations attributed to the SBS88 and ID18 mutational signatures with ASR across countries for colorectal cancer (**e**) and, independently, for colon and rectal cancers (**f**). Data points were coloured on the basis of signature prevalence, with size indicating the total number of cases per country. Statistically significant associations were evaluated using the sample-level multivariable linear regression models used in **d** (**e**) and similar models adjusted by age of diagnosis, sex and tumour purity (**f**). Blue lines and bands indicate univariate linear regressions and 95% confidence intervals for average signature activity versus ASR.

Despite the similar mutation profiles across countries (Extended Data Fig. 3), several signatures exhibited varying prevalence when comparing one country to all others (Fig. 2a, Supplementary Fig. 5 and Supplementary Table 16). Notably, SBS89 (OR = 28.0, *q* = 0.001), DBS8 (OR = 8.9, *q* = 3.2×10^−4^), and the novel ID_J (OR = 9.6, *q* = 6.2 × 10^−5^) were at higher frequencies in Argentina compared with all other countries (Fig. 2b). Signatures SBS89, DBS8 and ID_J also showed a strong tendency to co-occur (*P* < 1.7 × 10^−11^), suggesting they may arise from the same underlying mutational process. In Colombia (Fig. 2c), higher frequencies were observed for SBS94 (OR = 19.7, *q* = 3.2 × 10^−5^), the novel SBS_F (OR = 10.7, *q* = 2.0 × 10^−4^) and DBS6 (OR = 12.5, *q* = 0.028) compared with all other countries, with evidence of co-occurrence of SBS94 with SBS_F (*P* = 0.017) and DBS6 (*P* = 1.9 × 10^−4^). Enrichments were also found for SBS2 (OR = 2.0, *q* = 0.041) and SBS_H (OR = 2.3, *q* = 0.001) in Russia and CN_F (OR = 3.5, *q* = 3.9 × 10^−4^) in Brazil, whereas depletions were identified for DBS2 in Thailand (OR = 0.38, *q* = 0.008) and for DBS4 in Colombia (OR = 0.06, *q* = 0.034; Fig. 2a). Overall, the results indicate international differences in the prevalence of certain mutational processes involved in colorectal cancer development.

**Extended Data Fig. 6:**
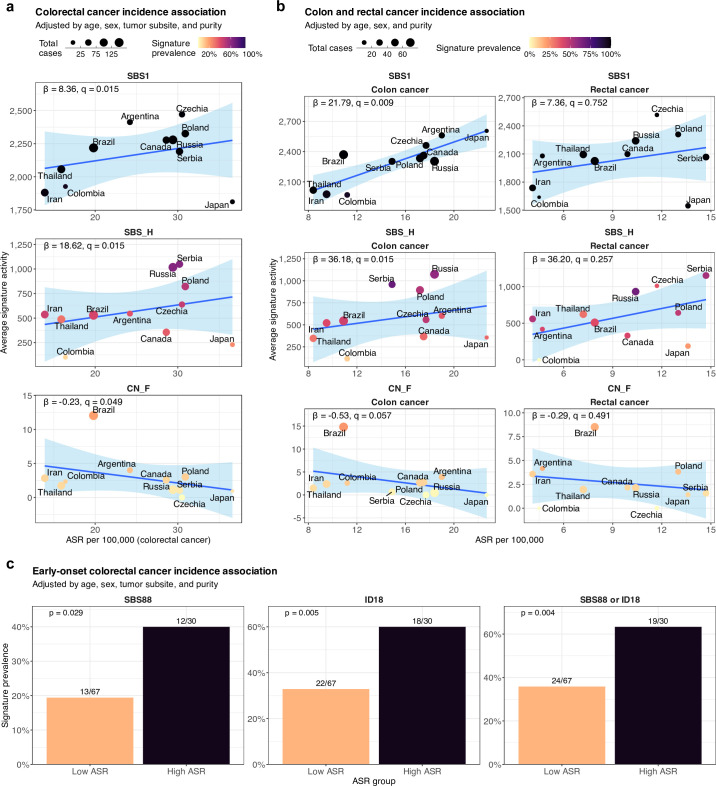
Association of mutational signatures with colorectal, colon, and rectal cancer incidence rates. **a-b**, Scatter plots indicating the association of the mutations attributed to signatures SBS1, SBS_H, and CN_F with the age-standardized incidence rates across countries for colorectal cancers (**a**), and independently for colon and rectal cancer (**b**). Data points were colored based on signature prevalence, with their size indicating the total number of cases per country. Statistically significant associations were evaluated using the sample-level multivariable linear regression models used in Fig. 2d (**a**), and similar multivariable linear regression models adjusted by age of diagnosis, sex, and tumor purity (**b**). Blue lines and bands indicate univariate linear regressions and 95% confidence intervals for average signature activity vs. ASR. **c**, Bar plots indicating mutational signature prevalence enrichment between low and high ASR countries (defined as those below or above an ASR of 7 per 100,000 people, for early-onset colorectal cancer, diagnosed between 20 and 49 years old). Statistically significant associations were evaluated using multivariable logistic regression models for early-onset colorectal cancer ASR adjusted by age of diagnosis, sex, tumor subsite, and tumor purity.

To explore the broader epidemiological implications of international variation in mutational processes, as previously done for kidney cancer^13^, we evaluated the relationships between ASR and mutational signatures (Fig. 2d and Supplementary Table 17). Independent of covariates, colibactin-induced mutational signatures, SBS88 and ID18, as well as clock-like signature SBS1 and novel signature SBS_H, associated with an increasing rate of ASR for colorectal cancer, whereas novel signature CN_F associated with a reduced ASR rate (*q* < 0.05; Fig. 2d,e and Extended Data Fig. 6a). For SBS88 and ID18, the association was linked with the ASR for rectal cancer (*q* = 0.088 and *q* = 0.008; Fig. 2f and Supplementary Table 18). By contrast, for SBS1, SBS_H and CN_F, the association was particularly strong for the ASR of colon cancer (*q* = 0.009, *q* = 0.015 and *q* = 0.057; Extended Data Fig. 6b). Colibactin-associated signatures were also found to be more prevalent in individuals from countries with high ASRs for early-onset colorectal cancer (Extended Data Fig. 6c).

## Enrichment of colibactin signatures

**Fig. 3:**
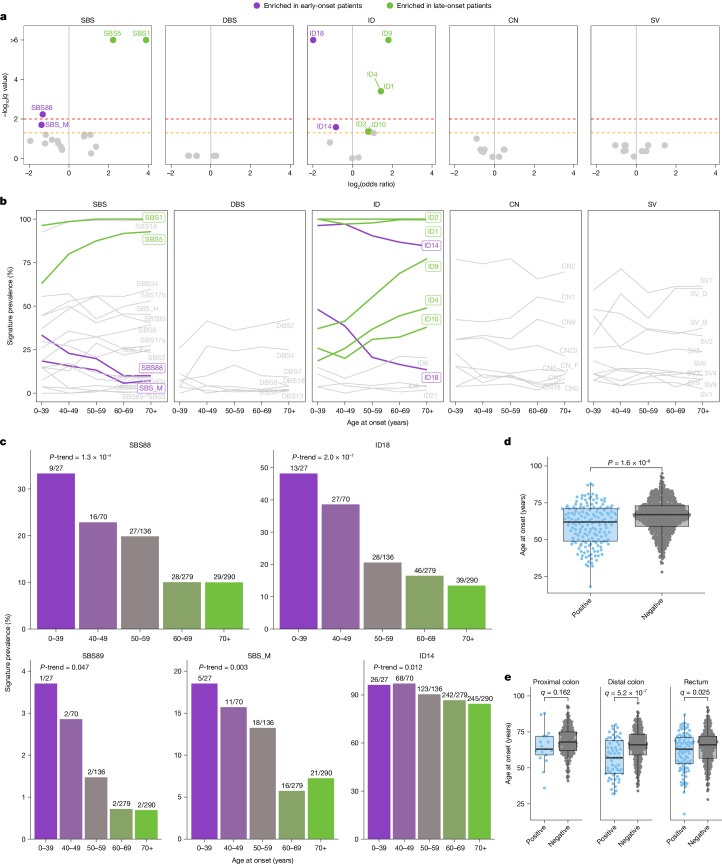
Variation of mutational signatures with age of onset in MSS colorectal cancers. **a**, Enrichment of signature prevalence in early-onset and late-onset cases. Statistical significance was evaluated using multivariable logistic regression models for age of onset categorized in two subgroups (less than 50 years of age and more than 50 years of age) and adjusted by sex, country, tumour subsite and tumour purity. Firth’s bias-reduced logistic regressions were used for regression presenting complete or quasi-complete separation. *P* values were adjusted for multiple comparisons using the Benjamini–Hochberg method based on the total number of mutational signatures considered per variant type and reported as *q* values. Dashed lines indicate *q* values of 0.05 (orange) and 0.01 (red). **b**, Signature prevalence trend across ages of onset. Signatures significantly enriched in early-onset or late-onset cases (from **a**) were coloured in purple and green, respectively. **c**, Signature prevalence across age groups. Statistically significant trends were evaluated using multivariable logistic regression models for age categorized in five subgroups (0–39, 40–49, 50–59, 60–69 and ≥70 years) and similar adjustments as in **a**, with Firth’s bias-reduced regressions for complete or quasi-complete separation cases. **d**,**e**, Age of onset variation according to the presence (*n* = 169) or absence (*n* = 633) of colibactin signatures (SBS88, ID18 or both) in all cases (**d**) and across tumour subsites, including proximal colon (*n* = 17, *n* = 172), distal colon (*n* = 61, *n* = 237) and rectum (*n* = 91, *n* = 224) (**e**). Statistically significant differences were evaluated using multivariable linear regression models adjusted by sex, country, tumour purity and tumour subsite (only for the analysis of all cases (**d**)). *P* values in **e** were adjusted for multiple comparisons based on the three tumour subsites considered and reported as *q* values. In box plots, the horizontal line indicates the median, the upper and lower ends of the box indicate the 25th and 75th percentiles. Whiskers show 1.5 × the interquartile range, and values outside the whiskers are shown as individual data points.

In addition to examining the global distribution of mutational signatures, the substantial number of early-onset colorectal cancer cases enabled evaluation of the association between mutational signatures and age at diagnosis. Although the average mutation profiles of early-onset and late-onset colorectal cancer cases were similar (Fig. 1e–g), the prevalence of some mutational signatures was associated with the age of diagnosis, independently of country of origin (Fig. 3a and Supplementary Table 19), genetic ancestry or ethnicity (Supplementary Figs. 6–8). As expected, late-onset cases showed enrichment in signatures that are known to accumulate linearly with age in normal colorectal crypts^35^, including SBS1, SBS5, ID1 and ID2 (Fig. 3a,b). The signatures of small IDs of unknown aetiology ID4, ID9 and ID10 also showed associations with late-onset cases (Fig. 3a,b).

**Extended Data Fig. 7:**
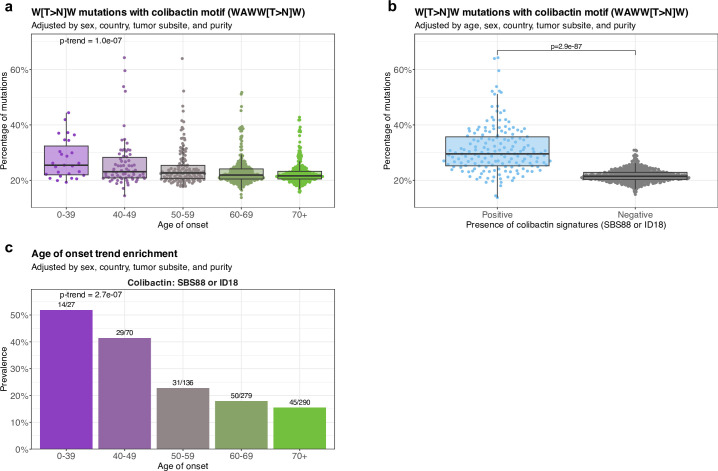
Enrichment of colibactin mutagenesis in early-onset colorectal cancers based on motif analysis. **a**, Box plots indicating the percentage of total W[T > N]W mutations with the WAWW[T > N]W motif across different age groups (0–39 *n* = 27, 40−49 *n* = 70, 50–59 *n* = 136, 60–69 *n* = 279, 70+ *n* = 290). Statistically significant trend was evaluated using a multivariable linear regression model adjusted by sex, country, tumor subsite, and tumor purity. The line within the box indicates the median, while the upper and lower ends indicate the 25^th^ and 75^th^ percentiles. Whiskers show 1.5 × interquartile range, and values outside are shown as individual data points. **b**, Box plots indicating the percentage of total W[T > N]W mutations with the WAWW[T > N]W motif across samples grouped by colibactin exposure status, determined by the presence (*n* = 169) or absence (*n* = 633) of signatures SBS88 or ID18. Statistical significance was evaluated using a multivariable linear regression model adjusted by age, sex, country, tumor subsite, and tumor purity. The line within the box indicates the median, while the upper and lower ends indicate the 25^th^ and 75^th^ percentiles. Whiskers show 1.5 × interquartile range, and values outside are shown as individual data points. **c**, Bar plots indicating the prevalence of colibactin exposure across age groups, with indication of the total number of cases where colibactin signatures were detected. Statistically significant trend was evaluated using a multivariable logistic regression model adjusted by sex, country, tumor subsite, and tumor purity.

By contrast, enrichment in early-onset cancers was observed for colibactin-induced signatures. Signatures SBS88 and ID18 were 2.5 and 4 times more common, respectively, in colorectal cancers diagnosed before 50 years of age than those diagnosed after (*q* = 0.006 and *q* = 3.7 × 10^−7^, respectively; Fig. 3a,b). The primary associations of early-onset cases with SBS88 and ID18 were further supported by the successive decline in the prevalence of these signatures with increasing age of diagnosis (*P* trend = 1.3 × 10^−4^ and *P* trend = 2.0 × 10^−7^, respectively; Fig. 3c and Supplementary Table 20). A similar effect was observed using a complementary motif enrichment analysis for detecting SBS88, similarly to a recent study^27^ (*P* trend = 1.0 × 10^−7^; Extended Data Fig. 7a,b). On the basis of the strong co-occurrence of SBS88 and ID18 (*P* = 7.4 × 10^−63^), as well as previous functional^7^ and population studies^6,23,27^, we defined exposure to colibactin by the presence of either SBS88 or ID18. Colibactin exposure was found in 21.1% of all MSS colorectal cancers (169 out of 802) and was associated with earlier age of onset (median age: 62 versus 67 years, *P* = 1.6 × 10^−8^; Fig. 3d), an effect more evident in the distal colon (median age: 57 versus 66 years, *q* = 5.2 × 10^−7^) and rectum (median age: 63 versus 66 years, *q* = 0.025; Fig. 3e). Overall, colibactin exposure had a strong inverse correlation with age, being 3.3 times more common in colorectal cancers diagnosed in individuals younger than 40 years compared to those over 70 years (*P* trend = 2.7 × 10^−7^; Extended Data Fig. 7c).

Signatures of unknown aetiology SBS_M and ID14 (Fig. 3a–c) were also enriched in early-onset cases, and SBS89 similarly exhibited a higher prevalence in younger individuals (5.8 times more prevalent in patients with early-onset compared with late-onset colorectal cancer; *P* trend = 0.047), albeit based on a very small number of cancers with the signature (9 out of 802, 1.1%; Fig. 3c). Notably, SBS_M showed an increase in distal colon and rectum tumours compared with proximal colon, similar to the one observed in colibactin-associated signatures SBS88 and ID18, previously reported^23^ (Supplementary Fig. 9).

## Colibactin is an early mutagenic event

**Fig. 4:**
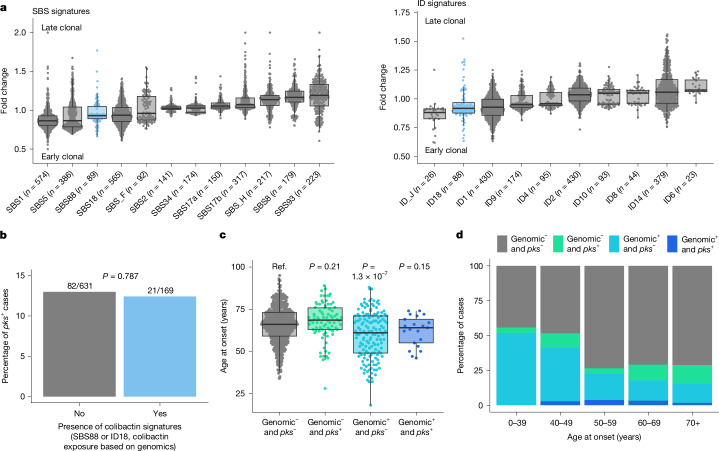
Colibactin mutagenesis as an early event in MSS colorectal cancer evolution. **a**, Fold change of the relative contribution per sample of each signature between early clonal and late clonal SBSs (left) and IDs (right). SBS signatures that contribute early and late clonal SBSs in fewer than 50 samples were excluded from the analysis. Similarly, ID signatures that contribute early and late clonal IDs in fewer than 20 samples were also excluded. Signatures were sorted by median fold change. **b**, Lack of concordance between colibactin exposure status determined by the presence of colibactin-induced signatures SBS88 or ID18, and the microbiome *pks* status. Statistical significance was evaluated using a multivariable Firth’s bias-reduced logistic regression model (due to quasi-complete separation) adjusted by age of diagnosis, sex, country, tumour subsite and tumour purity. **c**,**d**, Distribution of age of onset (**c**) and cases across age groups (**d**) based on the detection of colibactin-positive samples using genomic and microbiome status. The genomic status is defined by the presence of SBS88 or ID18; the microbiome status (*pks*) is determined by coverage of at least half of the *pks* island, and suggests ongoing or active *pks*^+^ bacterial infection (genomic^−^ *pks*^−^ *n* = 549, genomic^−^ *pks*^+^ *n* = 82, genomic^+^ *pks*^−^ *n* = 148, genomic^+^ *pks*^+^ *n* = 21). Statistical significance was evaluated using a multivariable linear regression model adjusted by sex, country, tumour subsite and tumour purity. In box plots, the horizontal line indicates the median, the upper and lower ends of the box indicate the 25th and 75th percentiles. Whiskers show 1.5 × the interquartile range, and values outside the whiskers are shown as individual data points.

**Extended Data Fig. 8:**
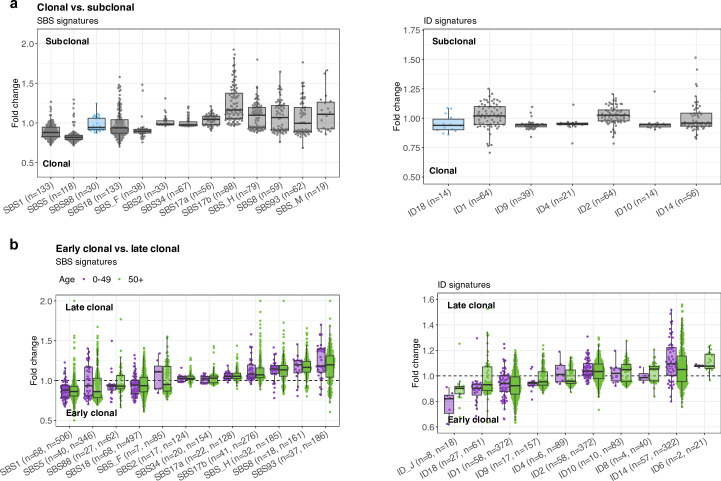
Enrichment of colibactin mutagenesis as an early clonal event in early-onset and late-onset colorectal cancers. **a**, Box plots indicating the fold-change of the relative contribution per sample of each signature between clonal and subclonal single base substitutions (SBS, left) and small insertions and deletions (ID, right). Signatures that generated clonal somatic mutations in fewer than 10 samples and also generated subclonal somatic mutations in fewer than 10 samples were excluded from the analysis. The line within the box indicates the median, while the upper and lower ends indicate the 25^th^ and 75^th^ percentiles. Whiskers show 1.5 × interquartile range, and values outside are shown as individual data points. **b**, Boxplots indicating the fold-change of the relative contribution per sample of each signature between early clonal and late clonal SBS (left) and ID (right) with samples separated by age of diagnosis in early-onset (under 50 years of age; purple) and late-onset (50 or over; green). As in Fig. 4a, SBS signatures that generated early clonal SBSs in fewer than 50 samples and late clonal SBSs in fewer than 50 samples, as well as ID signatures generating early clonal IDs in fewer than 20 samples and late clonal IDs in fewer than 20 samples, were excluded from the analysis.

To time the imprinting of SBS88 and ID18, mutations were categorized as early clonal, late clonal or subclonal during the development of each cancer and the contribution of each mutational signature to each category was determined (Methods). SBS88 and ID18 were both enriched in early clonal compared with late clonal mutations (*q* = 4.2 × 10^−4^ and *q* = 6.1 × 10^−5^; Fig. 4a), as well as a similar trend in clonal compared with subclonal mutations (*q* = 0.138 and *q* = 0.058; Extended Data Fig. 8a), consistent with the presence of these mutational signatures in normal colorectal epithelium^6^. This enrichment in earlier evolutionary stages was similar to the one observed for other well-known clock-like signatures such as SBS1, SBS5 or ID1 (Fig. 4a,b), as previously shown in tumours^36,37^ and normal tissues^6^, and in contrast to signatures that are known to preferentially generate late clonal and subclonal mutations, such as SBS17a or SBS17b^36^. Of note, the enrichment of colibactin signatures in early clonal mutations was observed for both early-onset (*q* = 0.004 for SBS88 and *q* = 2.0 × 10^−4^ for ID18) and late-onset colorectal cancer cases (*q* = 0.020 and *q* = 0.024; Extended Data Fig. 8b).

**Extended Data Fig. 9:**
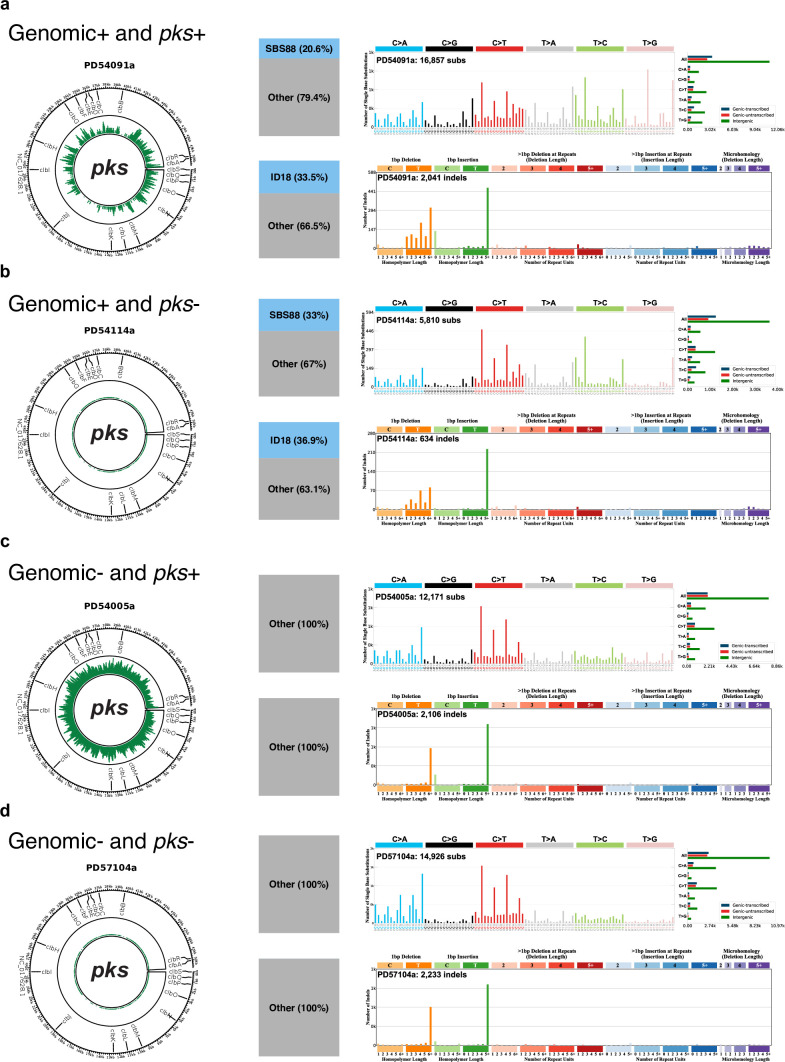
Representative microbiome and genomic profiles of colibactin-exposed samples. **a-d**, Microbiome and genomic profiles of representative samples corresponding to the four different sample types according to colibactin exposure: genomic+ and *pks* + (**a**), genomic+ and *pks−* (**b**), genomic- and *pks* + (**c**), and genomic- and *pks−*. The genomic status is defined by the presence of SBS88 or ID18 signatures, while the microbiome status (*pks*) is determined by the coverage of at least half of the *pks* island, and suggests ongoing and/or active *pks*+ bacterial infection. Circos plots display Reads Per Kilobase of transcript per Million (RPKM) values across *clb* genes within the *pks* island (left). Bar plots represent the proportion of mutations attributed to SBS88 and ID18 colibactin signatures compared to others (center), and are displayed next to mutational profiles of single base substitutions (SBS-288 mutational context) and small insertions and deletions (ID-83 mutational context) for each sample (right).

Since colibactin is produced by bacteria carrying the *pks* pathogenicity island, we investigated whether colorectal cancer cases with SBS88 or ID18 harboured *pks*^+^ bacteria based on sequencing reads from the cancer sample that did not map to the human genome but mapped to the *pks* locus (Methods). Consistent with a prior observation^38^, there was no association between the presence of SBS88 or ID18 and that of *pks*^+^ bacteria (Fig. 4b and Extended Data Fig. 9). Similarly, no microbiome association was observed for the other signatures enriched in early-onset colorectal cancers (Supplementary Note). Moreover, we observed a younger age of diagnosis for cases with SBS88 or ID18 but without an identified *pks*^+^ bacteria (*P* = 1.3 × 10^−7^; Fig. 4c,d). Although the reasons are unclear, one likely explanation is the imprinting of SBS88 and ID18 on the colorectal epithelium during an early period of life when *pks*^+^ bacteria were present, followed by the natural plasticity of the microbiome over subsequent decades, leading to the loss or gain of *pks*^+^ bacteria.

## Colibactin exposure and driver mutations

**Fig. 5:**
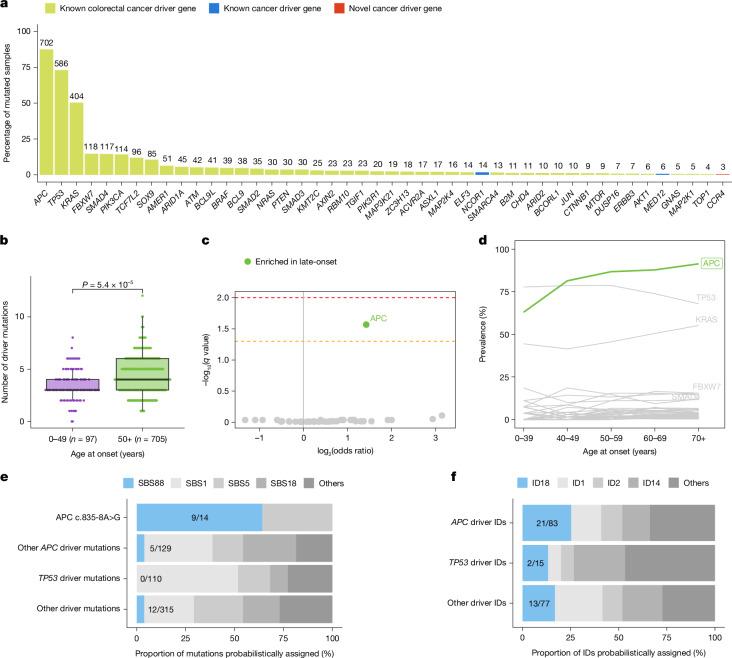
Variation of driver mutations with age of onset and association with colibactin mutagenesis in MSS colorectal cancers. **a**, Prevalence of driver mutations affecting the 48 detected driver genes. Genes were coloured according to their status as known cancer driver genes for colorectal cancer, known cancer driver genes for other cancer types or newly detected cancer driver genes. **b**, Distribution of total driver mutations across early-onset and late-onset tumours. Statistical significance was evaluated using a multivariable linear regression model adjusted by sex, country, tumour subsite and tumour purity. In box plots, the horizontal line indicates the median, the upper and lower ends of the box indicate the 25th and 75th percentiles. Whiskers show 1.5 × the interquartile range, and values outside the whiskers are shown as individual data points. **c**, Enrichment of driver mutations in cancer driver genes in early-onset and late-onset cases. Statistically significant enrichments were evaluated using multivariable logistic regression models adjusted by sex, country, tumour subsite and tumour purity. Firth’s bias-reduced logistic regressions were used for regressions presenting complete or quasi-complete separation. *P* values were adjusted for multiple comparisons using the Benjamini–Hochberg method based on the total number of cancer driver genes and reported as *q* values. Dashed lines indicate *q* values of 0.05 (orange) and 0.01 (red). **d**, Prevalence of driver mutations in cancer driver genes across ages of onset. Cancer driver genes significantly enriched in late-onset cases (as shown in **c**) were coloured in green. **e**,**f**, Proportion of driver mutations probabilistically assigned to colibactin-induced and other SBS (**e**) and ID (**f**) signatures. Driver mutations were divided into different groups, including *APC* c.835-8A>G splicing-associated driver mutation, as well as driver mutations affecting *APC*, *TP53* and other cancer driver genes.

Using the IntOGen framework^39^, 46 genes under positive selection were identified, with 8 being mutated in more than 10% of cancers: *APC*, *TP53*, *KRAS*, *FBXW7*, *SMAD4*, *PIK3CA*, *TCFL2* and *SOX9* (Fig. 5a and Supplementary Table 21). Forty-three out of the 46 genes have been previously reported as colorectal cancer driver genes^23,39^, two in other cancer types (*MED12* and *NCOR1*)^39^, and a putative novel colorectal cancer driver gene (*CCR4*) was identified with mutations indicating inactivation of the encoded protein. Mutations affecting these 46 cancer driver genes were annotated as driver mutations using a multi-step process on the basis of the mutation type and the mode of action of the gene (Methods). An increase in the total number of driver mutations was observed in late-onset compared to early-onset cases (FC = 1.21, *P* = 5.4 × 10^−5^; Fig. 5b). In addition, an enrichment in *APC* driver mutation carriers was also found for late-onset cases (OR = 2.7, *q* = 0.027; Fig. 5c,d and Supplementary Table 22), as previously reported^40^, whereas no hotspot driver mutations (defined as those affecting the same genomic position in at least 10 cases) were associated with age of onset (*q* > 0.05; Supplementary Table 23). No statistically significant differences across countries were found for driver mutations within cancer driver genes or for hotspot driver mutations (*q* > 0.05; Supplementary Tables 24 and 25).

**Extended Data Fig. 10:**
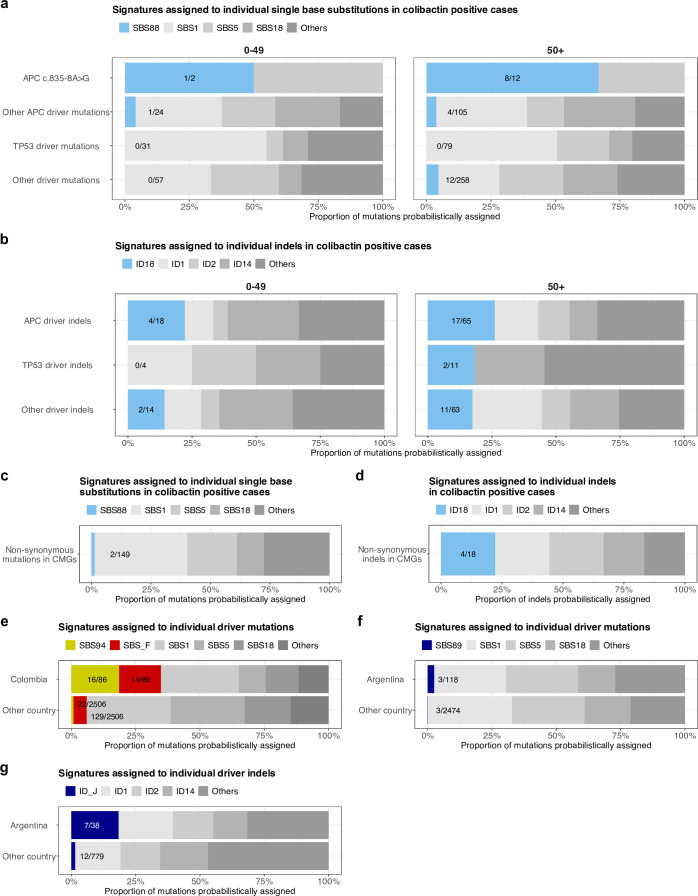
Driver mutations associated with colibactin mutagenesis in early-onset and late-onset colibactin positive cases and with country-enriched mutational signatures in microsatellite stable colorectal cancers. **a-b**, Bar plots indicating the proportion and number of driver mutations probabilistically assigned to colibactin-induced and other mutational signatures, including single base substitutions (**a**) and small insertions and deletions (indels; **b**), with samples separated by age of diagnosis in early-onset (under 50 years of age; left) and late-onset (50 or over; right). Driver mutations were divided into different groups, including the *APC* c.835−8 A > G splicing-associated driver mutation, other *APC* driver mutations, *TP53* driver mutations, and driver mutations affecting other cancer driver genes. **c-d**, Bar plots indicating the proportion and number of mutations in chromatin modifier genes probabilistically assigned to colibactin-induced and other mutational signatures, including single base substitutions (**c**) and indels (**d**), in the 169 colibactin positive cases. **e-g**, Bar plots indicating the proportion and number of driver single base substitutions (**e** and **f**) and indels (**g**) in cancer driver genes probabilistically assigned to specific mutational signatures in cases from Colombia (**e**) or Argentina (**f** and **g**) compared to other countries.

The contributions of SBS88 and ID18 to driver mutations were assessed using probabilistic assignment of signatures to individual mutations^41^. SBS88 accounted for 64.3% of the colibactin-induced^42^ *APC* splicing variant c.835-8A>G in colibactin-exposed samples, compared with only 3.9% and 3.8% of driver substitutions in *APC* or other cancer genes (Fig. 5e). Similarly, ID18 accounted for 25.3% of *APC* driver IDs and 16.9% of other driver IDs in colibactin-exposed cases (Fig. 5f). Overall, SBS88 and ID18 accounted for 8.3% of all SBS and ID driver mutations, and 15.5% of all *APC* driver mutations in colibactin-positive cancers. Nevertheless, no differences were observed between early-onset and late-onset colibactin-positive colorectal cancer in the proportion of driver mutations assigned to specific mutational signatures (Extended Data Fig. 10a,b). In addition, a prior study observed that SBS88 is also responsible for mutations in chromatin modifier genes^38^, and we were able to validate this as well as show a similar effect for the colibactin-associated ID signature, ID18 (Extended Data Fig. 10c,d). Of note, using a similar methodology, we observed an elevated number of driver mutations assigned to SBS94 and SBS_F in Colombia, as well as SBS89 and ID_J in Argentina, compared with other countries (Extended Data Fig. 10e–g).

## Discussion

Over the past seven decades, colorectal cancer incidence rates have shown complex changes with marked international variation. Notably, although many high-income countries have seen decreases in overall incidence rates, there has been an increase among adults below 50 years of age. If these trends continue into older age groups, they could reverse the current overall decline in colorectal cancer incidence. In this study, whole-genome sequences of 981 colorectal cancers from 11 countries revealed evidence of geographic and age-related variation in their landscapes of somatic mutation, which may contribute to explaining these global trends. These variations were found almost exclusively in the 802 microsatellite-stable colorectal cancers. For colorectal cancers with MSI, we observed limited geographic or age-related differences, possibly owing to the smaller sample size and the predominance of somatic mutations resulting from defective DNA repair mechanisms. Similarly, no differences were noted in colorectal cancers harbouring other DNA repair deficiencies.

For MSS colorectal cancers, the prevalence of certain mutational signatures was higher in some countries compared with all others, notably SBS89, DBS8 and ID_J in Argentina, and SBS94, SBS_F and DBS6 in Colombia. Although such geographic variation could, in principle, be due to differences in population-specific inheritance, it is more plausible that these are due to differences in exogenous environmental or lifestyle mutagenic exposures. Indeed, aside from country of origin, we also assessed the variability with genetic ancestry and self-reported ethnicity (Methods), although the homogenous distribution of these characteristics within countries (Supplementary Fig. 10) precluded us from clarifying whether the varying prevalence of signatures in different countries was related to genetic or environmental factors. The natures of the putative exposures underlying SBS89/DBS8/ID_J and SBS94/SBS_F/DBS6 are currently unknown. However, SBS89 shares several features with the colibactin-induced signatures SBS88 and ID18. SBS89 has been previously found in normal colorectal crypts^6^ but not in other normal cells. In individuals with SBS89, some crypts have these mutations whereas others do not. SBS89 appears to be imprinted on the normal colorectal epithelium early in life, with mutagenesis ceasing thereafter^6^. Moreover, SBS89 mutations show transcriptional strand bias^6^, a common trait of mutations caused by exogenous mutagenic exposures that form bulky covalent DNA adducts. Thus, SBS89 may also be caused by a mutagen originating from the colorectal microbiome and it is conceivable that multiple microbiome-derived mutagens may contribute to the mutation burden of the colorectal epithelium. Although the impact of country-specific microbiome-derived exposures on geographic differences in colorectal cancer incidence remains unclear, the correlations between colorectal cancer ASR and signatures SBS88 and ID18 suggest that microbiome-derived colibactin exposure may influence colorectal cancer incidence rates. Nonetheless, further studies are necessary to thoroughly investigate this hypothesis.

The evidence for enrichment of SBS88 and ID18 mutation burdens in early-onset colorectal cancers may indicate a role for colibactin exposure in the increase in early-onset colorectal cancer incidence over the past 20 years. Prior studies have indicated that mutagenesis due to colibactin exposure can occur within the first decade of life and then ceases^6^. In some instances, the mutation burden caused by this early-life mutation burst can endow affected colorectal crypts with the equivalent of decades of mutation accumulation and this ‘head start’ could thus plausibly result in an increased risk of early-onset cancers. One mechanism by which colibactin-induced mutagenesis might contribute to colorectal neoplastic change is by somatically inactivating one copy of *APC* through the generation of protein-truncating driver mutations. Since *APC* mutations usually occur early in the sequence of driver mutations leading to colorectal cancer^36,43^, a first-hit inactivating mutation in *APC* during early life could put an individual several decades ahead for developing colorectal cancer and resulting in a higher likelihood of early-onset colorectal cancer. The mutation profile of SBS88, with its preponderance of T>C substitutions, is intrinsically ineffective in generating translation termination codons, and SBS88 accounts for only a small proportion of *APC* driver base substitutions. However, colibactin mutagenesis entails a relatively high proportion of ID mutations, with the characteristic profile of ID18, almost all of which will introduce translational frameshifts in coding sequences. ID18 accounts for approximately one quarter of *APC* ID drivers in colibactin-positive cancers and is increased among *APC* ID drivers compared with ID drivers in other cancer genes such as *TP53*, which occur later in the multi-step process of colorectal carcinogenesis^44^. Thus colibactin-induced ID driver mutations in *APC* may account for a substantial proportion of any putative effect of colibactin on colorectal carcinogenesis. Conversely, the unexpected increase in driver mutations observed in late-onset colorectal cancers might suggest that we failed to identify all driver mutational events in early-onset cases, possibly overlooking additional effects of colibactin or other mutagenic exposures, and potentially related to alterations beyond *APC*, as early-onset cases are enriched in *APC* wild-type tumours^40^. In this context, body mass index, diet, lifestyle and other exposomal factors—particularly in early life—may have an important mutagenic role, with the lack of analyses on these factors being a limitation of the current study.

Although our results identify an association between the presence of colibactin-induced mutational signatures and early-onset colorectal cancer, complementing the prior finding that tumours harbouring colibactin mutagenesis have a younger average age at diagnosis^27^, further research is required to establish causality. Future studies should examine the SBS88 and ID18 mutation burdens of normal colorectal crypts from individuals with early-onset colorectal cancer (cases) and age-matched healthy individuals (controls) with the expectation of an enrichment in cancer cases if colibactin mutagenesis is causally implicated. If so, the increase in early-onset colorectal cancer over the past 30 years would indicate that an increased exposure to colibactin in affected populations occurred during the second half of the twentieth century, perhaps owing to increasing prevalence of *pks*^+^ bacteria, and genome sequences of appropriately selected colorectal cancers and normal colorectal tissues would inform on this historical flux. These studies could be supported by international and, if possible, retrospective studies of the prevalence of colibactin-producing *pks*^+^ bacteria in the colorectal microbiome, which should include paired stool samples or other methods for robust microbiome analysis, which were not available for the current study. Finally, reduced cancer incidence as a result of prevention of exposure to colibactin-producing bacteria during early life would provide definitive evidence of a causal role for colibactin in early-onset colorectal carcinogenesis.

In summary, mutational epidemiology reveals country-specific and age-specific variations in the prevalence of certain mutational signatures. The results also highlight the potential role of the large intestine microbiome as an early-life mutagenic factor in the development of colorectal cancer.

## Methods

### Recruitment of patients and informed consent

The International Agency for Research on Cancer (IARC/WHO) coordinated case recruitment through an international network of 17 collaborators from 11 participating countries in North America, South America, Asia and Europe (Supplementary Table 26). The inclusion criteria for patients were ≥18 years of age (ranging from 18 to 95, with a mean of 64 and a standard deviation of 12), confirmed diagnosis of primary colorectal cancer, and no prior treatment for colorectal cancer. Informed consent was obtained for all participants. Patients were excluded if they had any condition that could interfere with their ability to provide informed consent or if there were no means of obtaining adequate tissues or associated data as per the protocol requirements. Ethical approvals were first obtained from each Local Research Ethics Committee and Federal Ethics Committee when applicable, as well as from the IARC/WHO Ethics Committee.

### Bio-samples and data collection

Dedicated standard operating procedures, following guidelines from the International Cancer Genome Consortium (ICGC), were designed by IARC/WHO to select appropriate case series with complete biological samples and exposure information^45^, as described previously^11,13,14^ (Supplementary Table 26). In brief, for all case series included, anthropometric measures were taken, together with relevant information regarding medical and familial history. All biological samples from retrospective cohorts were collected using rigorous, standardized protocols and fulfilled the required standards of sample collection defined by the IARC/WHO for sequencing and analysis. Potential limitations of using retrospective clinical data collected using different protocols from different populations were addressed by a central data harmonization to ensure a comparable group of exposure variables (Supplementary Table 26). All patient-related data were pseudonymized locally through the use of a dedicated alpha-numerical identifier system before being transferred to the IARC/WHO central database. REDCap^46^ was used to collect epidemiological data.

### Expert pathology review

Original diagnostic pathology departments provided diagnostic histological details of contributing cases through standard abstract forms, together with a representative haematoxylin and eosin-stained slide of formalin-fixed paraffin-embedded tumour tissues whenever possible. IARC/WHO centralized the entire pathology workflow and coordinated a centralized digital pathology examination of the frozen tumour tissues collected for the study as well as formalin-fixed paraffin-embedded sections when available, via a web-based approach and dedicated expert panel following standardized procedures as described previously^11,13^. A minimum of 50% viable tumour cells was required for eligibility for whole-genome sequencing. In summary, frozen tumour tissues were first examined to confirm the morphological type and the percentage of viable tumour cells. A random selection of tumour tissues was independently evaluated by a second pathologist. Enrichment of tumour component was performed by dissection of the non-tumoural part, if necessary.

### DNA extraction

A total of 1,977 patients with primary colorectal cancer were enrolled into the study, including biological samples for 1,946 cases and sequencing data (FASTQ) for 31 cases from Japan. Of these, 906 samples (45.8%) were excluded due to insufficient viable tumour cells (pathology level) or inadequate DNA (tumour or germline). Extraction of DNA from fresh frozen primary tumour and matched blood/normal tissue samples was centrally conducted at IARC/WHO (except for samples from Japan) following a similarly standardized DNA extraction procedure. Germline DNA was extracted from whole blood (*n* = 1,015), except for a small subset of Canadian cases (*n* = 25) where only adjacent normal tissue was available, following previously described protocols and methods^11,13^. As a result, DNA from 1,040 individuals was sent to the Wellcome Sanger Institute for whole-genome sequencing.

### Whole-genome sequencing

Fluidigm SNP genotyping with a custom panel was performed to ensure that each pair of tumour and matched normal samples originated from the same individual. Whole-genome sequencing (150 bp paired-end) was performed on the Illumina NovaSeq 6000 platform with a target coverage of 40× for tumours and 20× for matched normal tissues. All sequencing reads were aligned to the GRCh38 human reference genome using the Burrows–Wheeler Aligner MEM (BWA-MEM; v0.7.16a and v0.7.17)^47^. Post-sequencing quality control metrics were applied for total coverage, evenness of coverage, contamination, and total number of somatic SBSs. Cases were excluded if coverage was below 30× for tumour or 15× for normal tissue. For evenness of coverage, the median over mean coverage (MoM) score was calculated. Tumours with MoM scores outside the range of values determined by previous studies^48^ to be appropriate for whole-genome sequencing (0.92–1.09) were excluded. Conpair^49^ was used to detect contamination, cases were excluded if the result was greater than 3%^48^. Finally, samples with <1,000 total somatic SBSs were also excluded. A total of 981 pairs of colorectal cancer and matched normal tissue passed all criteria. Comparing the clinicopathological characteristics between the included and excluded patients revealed very similar traits (Supplementary Table 27), and comparable to those expected for each country according to GLOBOCAN metrics (obtained from https://gco.iarc.who.int/today/en/dataviz/; Supplementary Fig. 11).

### Germline variant calling

Germline SNVs and IDs were derived from whole-genome sequencing from the normal paired material for each individual using Strelka2 with appropriate quality control criteria^50^. Variant calls were then derived into genotypes for each individual and annotated using ANNOVAR^51^.

### Somatic variant calling

Variant calling was performed using the standard Sanger bioinformatics analysis pipeline (https://github.com/cancerit). Copy number profiles were determined using ASCAT^52^ and BATTENBERG^53^ when tumour purity allowed. SNVs were called with cgpCaVEMan^54^, IDs were called with cgpPINDEL^55^, and structural rearrangements were called using BRASS (https://github.com/cancerit/BRASS). CaVEMan and BRASS were run using the copy number profile and purity values determined from ASCAT when possible (complete pipeline, *n* = 916). When tumour purity was insufficient to determine an accurate copy number profile (partial pipeline, *n* = 31) CaVEMan and BRASS were run using copy number defaults and an estimate of purity obtained from ASCAT. Finally, for a subset of cases which had no large CNs (copy number normal pipeline, *n* = 34), CaVEMan and BRASS were run using copy number defaults and an estimate of purity calculated by the median variant allele frequency (VAF) of IDs multiplied by two. For SNVs, additional filters on ASRD (read length-adjusted alignment score of reads showing the variant allele) and CLPM (median number of soft-clipped bases in variant supporting reads; ASRD ≥ 140 and CLPM = 0) were applied in addition to the standard PASS filter to remove potential false positive calls. To further exclude the possibility of caller-specific artefacts being included in the analysis, a second variant caller was run, Strelka2^50^ for SNVs and Manta^56^ for IDs. Only variants called by both the Sanger variant calling pipeline and Strelka2/Manta were included in subsequent analysis.

### Generation of mutational matrices

Mutational matrices for SBSs, IDs, DBSs, CNs and SVs were generated using SigProfilerMatrixGenerator with default options (v1.2.0)^57,58^.

### MSI validation

The presence of MSI in colorectal cancers was validated using the QX200 Droplet Digital PCR System (Bio-Rad) for the detection of five microsatellite markers (BAT25, BAT26, NR21, NR24 and Mono27) commercially pooled in three primer–probe mix assays, as previously described^59^. In brief, samples were tested in duplicate, and each reaction comprised 1× ddPCR Multiplex Supermix for probes (Bio-Rad), 1× primer–probe mix and 10 ng of extracted tumour DNA, in a total volume of 22 μl. MSI-positive, negative and no-template (nuclease-free water) controls were included in each experiment. Droplet generation and plate preparation for thermal cycling amplification were performed using the QX200 AutoDG Droplet Digital PCR System (Bio-Rad). The following PCR protocol was applied on a C1000 Touch Thermal Cycler (Bio-Rad): 37 °C for 30 min, 95 °C for 10 min, followed by 40 cycles of denaturation at 94 °C for 30 s, annealing at 55 °C for 1 min, with a final extension at 98 °C for 10 min. Following PCR amplification, fluorescence signals were quantified using the QX200 Droplet Reader (Bio-Rad), and data were analysed with QuantaSoft Analysis Pro v1.0.596.0525 (Bio-Rad) software. Positive and negative controls served as guides to call markers and delineate clusters. For each assay, the cluster at the bottom left of the *xy* plot was designated as the negative population. Clusters located vertically and horizontally from the negative cluster were identified as the mutant population, while clusters located diagonally from the negative cluster represented the wild-type population. Tumours were characterized for the MSI phenotype by analysing the results for all five markers using the following criteria: MSI-positive if two or more mutant microsatellite markers were observed, and MSS (that is, MSI-negative) when none or only one of the microsatellite markers was altered (Supplementary Note and Supplementary Table 28).

### Extraction of mutational signatures

Mutational signatures were primarily extracted using SigProfilerExtractor^34^, based on non-negative matrix factorization, and validated by mSigHdp^60^, based on hierarchical Dirichlet process mixture models.

For SigProfilerExtractor (v1.1.21), de novo mutational signatures were extracted from SBS, DBS and ID mutational matrices using 500 NMF replicates (nmf_replicates=500), nndsvd_min initialization (nmf_init = “nndvsd_min”), and default parameters. Extractions were performed separately on the subsets of 802 MSS and 153 MSI cases (Supplementary Tables 5–9 and 29–33). De novo SBS mutational signatures were extracted for both SBS-288 and SBS-1536 contexts, which, beyond the common SBS-96 trinucleotide context using the mutated base and the 5′ and 3′ adjacent nucleotides^57,61^, also consider the transcriptional strand bias and the pentanucleotide context (two 5′ and 3′ adjacent nucleotides), respectively. SBS-288 extends the SBS-96 contexts by classifying mutations into transcribed, untranscribed, or intergenic non-transcribed regions, whereas SBS-1536 considers the two flanking bases on either side of the mutated base to form a pentanucleotide context^57^. In MSS colorectal tumours, using the SBS-288 and SBS-1536 contexts 16 and 14 signatures were extracted, respectively (Supplementary Fig. 12). In order to calculate the cosine similarity, the SBS-288 and SBS-1536 signatures were both collapsed into the SBS-96 mutational context. Fourteen signatures were extracted in both formats with cosine similarity >0.9 (Supplementary Fig. 12 and Supplementary Table 34). The two signatures extracted only in the SBS-288 format were SBS_E and SBS_K. The former is a flat signature that decomposed to SBS1, SBS3 and SBS5, and the latter represents an incomplete separation of SBS17a and SBS17b (Supplementary Table 10). The SBS-288 signature results were used for further analysis as this format allowed the extraction of these additional signatures (Supplementary Tables 5 and 29). Notably, all four MSS signatures where decomposition was rejected (SBS_F, SBS_H, SBS_M and SBS_O; Supplementary Table 10) could be reproduced in both SBS-288 and SBS-1536 formats with a cosine similarity >0.95 (Supplementary Table 34).

Previously established mutational contexts DBS-78 and ID-83^21,57^ were used for the extraction of DBS and ID signatures (Supplementary Tables 6, 7, 30 and 31). Copy number signatures were extracted de novo using SigProfilerExtractor with default parameters and following an updated context definition benefitting from WGS data (CN-68) (Supplementary Tables 8 and 32), which allowed to further characterize CN segments below 100 kb in length (in contrast to current COSMICv3.4 reference signatures using the CN-48 context, which were based on SNP6 microarray data and therefore without the resolution to characterize short CN segments)^62^. SV signatures were extracted using a similarly refined context, with an in-depth characterization of short SV alterations below 1 kb (SV-38 context, in contrast to current COSMICv3.4 signatures based on the SV-32 context^63^; Supplementary Tables 9 and 33).

mSigHdp^60^ extraction of SBS-96 and ID-83 signatures was performed on the 802 MSS subset to validate the mutational signatures obtained using SigProfilerExtractor using the suggested parameters and using the country of origin to construct the hierarchy. SigProfilerAssignment was subsequently used to match mSigHdp de novo signatures to previously identified COSMICv3.4 signatures. SBS extraction was performed using the SBS-96 mutational context only, as mSigHdp has not been benchmarked for extended contexts. In total, mSigHdp extracted 17 signatures (Supplementary Fig. 13), of which 15 were very close matches (cosine similarity >0.90) to the SBS-288 results, whereas signatures hdp.14 and hdp.17 were unique to mSigHdp (Supplementary Table 34). The first unique signature, hdp.14, was an additional APOBEC containing signature, whereas hdp.17 was a combination of the new signature hdp.10 (SBS_H) and SBS93 (hdp.7/SBS_I). The latter was confirmed by performing a decomposition using the panel of COSMICv3.4 and the new colorectal cancer signatures as the signature database. Again, all four signatures where decomposition was rejected (SBS_F, SBS_H, SBS_M and SBS_O) could be reproduced in mSigHdp with a cosine similarity >0.95 (Supplementary Table 34), indicating that these signatures are highly reproducible using independent methodology. For ID-83 signatures, mSigHdp extracted eight signatures in comparison to the ten extracted from SigProfilerExtractor (Supplementary Fig. 14). Of these, six mSigHdp signatures could be matched directly to those from SigProfilerExtractor (Supplementary Table 35). Of the remaining two mSigHdp signatures, hdp.6 appears to be a combination of the SigProfilerExtractor de novo signatures ID_G and ID_H (but missing the ID1 component of both of those de novo signatures), whereas hdp.8 is a combination of ID2, ID5, and ID9. ID5 is not included in the final panel of signatures decomposed from the SigProfilerExtractor (Extended Data Fig. 4d,e and Supplementary Table 10) and was not included in the final panel of signatures used for signature assignment (Supplementary Table 12). Notably, the novel signature ID_J (Extended Data Fig. 4e) was reproducible in mSigHdp but with low cosine similarity (0.64; Supplementary Table 35). However, the low cosine similarity is explained by the lack of ID1 contamination in the signature extracted using mSigHdp.

### Decomposition of mutational signatures

After de novo extraction was completed, SigProfilerAssignment^41^ v0.0.29 was used to decompose the de novo extracted SBS, ID, DBS, CN and SV mutational signatures into COSMICv3.4 reference signatures based on the GRCh38 reference genome^64^ (Supplementary Tables 10 and 36). When possible, SigProfilerAssignment matched each de novo extracted mutational signature to a set of previously identified COSMICv3.4 signatures. For the SBS-288, CN-68 and SV-38 signatures, this required collapsing the high-definition classifications into the standard SBS-96, CN-48 and SV-32 mutational classifications, respectively. As a result of the loss of information from extended contexts and also due to the large number of COSMICv3.4 reference mutational signatures, it is likely that the decompositions on default settings will include signatures that are implausible given the cancer type.

For the MSS SBS signatures, using the default decomposition settings, only SBS_M was not decomposed. In order to optimize the decomposition, the following signature subgroups were excluded (using the exclude_signature_subgroups parameter of SigProfilerAssignment): artefact signatures, ultraviolet signatures, lymphoid signatures, mismatch repair deficiency signatures, polymerase deficiency signatures, base excision repair deficiency signatures, and treatment signatures. In addition, the new_signature_threshold was set to 0.90. Using these settings, SBS_H and SBS_O, in addition to SBS_M, were not decomposed. SBS_F was still decomposed into SBS1, SBS5, SBS19 and SBS40a in the optimized decomposition. However, this was rejected on the basis that SBS_F had been previously extracted in an independent cohort^22^ and the lack of individual spectra that supported the presence of SBS19 (the other reference SBS signatures all appeared in other decompositions). By contrast, SBS_D, despite being a borderline signature, with a cosine similarity of 0.90, had not previously been extracted in independent cohorts. Therefore, we chose to be conservative and not consider SBS_D as a novel signature. As such, SBS_D was decomposed into SBS1, SBS5, SBS18, and SBS34 (Supplementary Table 10). Regarding other non-decomposed signatures, SBS_H and SBS_M also showed a strong similarity with previously reported signatures in the UK population^22^ (cosine similarity >0.94), whereas SBS_O reflected a cleaner version of a previously reported COSMICv3.4 signature (SBS41). To validate the latter, we performed a decomposition of the current mutational profile of signature SBS41 using the decomposed signatures from our analysis, obtaining a confirmation that SBS41 can be reconstructed by a linear combination of SBS_O (contributing 19.00% of the mutational profile), SBS93 (62.54%), SBS34 (12.60%) and SBS5 (5.86%) with a cosine similarity of 0.91. Notably, SBS93, first identified in gastric tumours^34^, was unknown at the time SBS41 was first reported^21^.

For the MSI SBS extractions, using default settings for SigProfilerAssignment, only SBS_M_MSI was not decomposed, also showing a strong similarity with a previously reported signature in the UK population^22^ (cosine similarity = 0.89). In order to optimize the decomposition, the following signature subgroups were excluded (using the exclude_signature_subgroups parameter): artefact signatures, ultraviolet signatures, lymphoid signatures, polymerase deficiency signatures, base excision repair deficiency signatures, homologous repair deficiency signatures and treatment signatures. Optimizing did not change the number of signatures that were not decomposed. However, the decompositions for SBS_I_MSI, SBS_N_MSI and SBS_O_MSI were subsequently rejected on the basis that individual spectra existed that strongly support these signatures being the result of distinct mutational processes (Supplementary Fig. 15 and Supplementary Table 36).

Regarding other variant types (using similar parameters as previously mentioned for SBS signatures), for the MSS cohort, one ID (ID_J), one CN (CN_F) and two SV signatures (SV_B and SV_D) were additionally not decomposed into previously known signatures, and therefore considered as novel (Supplementary Table 10). The novel SV signature SV_D, identified in the MSS cohort, was also considered for the decomposition of de novo SV signatures extracted in the MSI cohort. In the MSI cohort, one de novo DBS signature (DBS_B_MSI) did not match any COSMICv3.4 signatures and was considered as novel (Supplementary Table 36).

### Attribution of mutational signatures to individual samples

Known COSMIC signatures and de novo signatures that were not decomposed into COSMIC signatures (Supplementary Tables 10 and 36) were attributed for each sample using MSA^65^ (v2.0) for SBS, ID, and DBS, whereas SigProfilerAssignment^41^ was used for CN and SV (Supplementary Tables 11–15 and 37–41). A conservative approach was used for MSA attributions utilizing the (params.no_CI_for_penalties=False) option for the calculation of optimum penalties. Pruned attributions were used for the final analysis, where confidence intervals were applied to each attributed mutational signature and any signature activity with a lower confidence limit equal to 0 was removed.

### Attribution of mutational signatures to individual somatic mutations

SBS and ID mutational signatures were probabilistically attributed to individual somatic mutations using the MSA activities per sample, based on Bayes’ rule and the specific mutational context for the mutation, as previously described^41^. In brief, to calculate the probability of a specific mutational signature being responsible for a mutation in a given mutational context and in a particular sample, we multiplied the general probability of the signature causing mutations in a specific mutational context (obtained from the mutational signature profile) by the activity of the signature in the sample (obtained from the signature activities), and then normalized this value dividing by the total number of mutations corresponding to the specific mutational context (obtained from the reconstructed mutational profile of the sample). The signature with the maximum likelihood estimation was assigned to each individual somatic mutation.

### Quantification of DNA repair deficiency-associated mutational signatures in DNA repair-deficient cases

To quantify the number of mutations contributed by signatures associated with DNA repair deficiencies in the 24 cases classified as DNA repair-deficient, we assigned directly all SBS and ID COSMICv3.4 signatures^64^, as well as Signal SBS108^22^ (related to *OGG1* deficiency) to these samples using SigProfilerAssignment^41^. The following SBS signatures were considered to quantify the mutations contributed by *MUTYH* mutations (COSMIC SBS36; Supplementary Fig. 2b), *NTHL1* mutations (COSMIC SBS30; Supplementary Fig. 2d), *OGG1* mutations (Signal SBS108; Supplementary Fig. 2f), *POLD1* mutations (COSMIC SBS10c; Supplementary Fig. 2h) and *POLE* mutations (COSMIC SBS10a, SBS10b and SBS28; Supplementary Fig. 3b), whereas COSMIC ID signature ID6 was considered to characterize the IDs contributed by homologous recombination deficiency (Supplementary Fig. 1b).

### Sensitivity analysis for the detection of colibactin signatures in MSS tumours

To assess the resolution for detecting colibactin signatures in MSS cases, we performed simulations for both SBSs and IDs. Specifically, synthetic SBS88 and ID18 mutations were injected at different average levels in each sample (scenarios 1%, 5%, 10%, 15% and 20% for SBS88, and 6%, 10%, 15%, 20% and 25% for ID18) for all MSS cases where the two colibactin-associated signatures were not originally detected. Mutations were injected according to a Gaussian distribution where the mean was equal to a percentage of a sample’s total mutational burden, and the standard deviation was equal to 10% of the mean. Importantly, the overall mutational burden for each sample was kept the same by randomly subtracting the same number of mutations that were injected into the sample, while ensuring all mutation counts were still non-negative. Mutational signatures were re-extracted as done for the original data, and MSA attributions were performed using the same penalties applied for the original data. In the SBS context, our analysis indicates that among the non-SBS88 positive cases in the original data (693 out of 802), SBS88 was attributed to samples if it contributes at least 2.5% of mutations (median of 0.025 relative proportion at the level of 1% injection; Supplementary Fig. 16a). Indeed, MSA attributed SBS88 in about 90.6% of simulation trials at the 1% injection level, approximately 99.7% at the 5% injection level, and 100% at the 10%, 15% and 20% injection levels. For the ID context, the results indicate that among the non-ID18 positive cases in the original data (649 out of 802), ID18 was attributed to samples if it contributed at least 6.6% of mutations (Supplementary Fig. 16b). MSA attributed ID18 in about 99.8% of simulation trials at the 6% injection level, and 100% at 10%, 15%, 20% and 25% injection levels. These results suggest that our analyses are unlikely to have overlooked SBS88 and ID18 in the examined set of MSS colorectal cancers, assuming they contribute at least 1% and 6% of mutations per sample, respectively.

### Driver gene analysis

Consensus de novo driver gene identification was performed by IntOGen^39^, which combines seven state-of-the-art computational methods to detect signals of positive selection across the cohort. The genes identified as drivers with a combination *q* value < 0.10 were classified according to their mode of action in tumorigenesis (that is, tumour suppressor genes or oncogenes) based on the relationship between the excess of observed nonsynonymous and truncating mutations computed by dNdScv^66^ and their annotations in the Cancer Gene Census^67^.

To identify potential driver mutations, we selected SBS or ID mutations that fulfilled any of the following criteria: mutations classified as ‘oncogenic’ or ‘likely oncogenic’ by OncoKB^68^ (annotated with OncoKB-annotator; https://github.com/oncokb/oncokb-annotator); mutations classified as drivers in the TCGA MC3 drivers study^69^; truncating mutations in driver genes annotated as tumour suppressors; recurrent missense mutations (seen in at least three cases); mutations classified as ‘likely drivers’ by boostDM (score >0.50)^70^; or missense mutations classified as ‘likely pathogenic’ by AlphaMissense^71^ in driver genes annotated as tumour suppressors. Six of the IntOGen-identified driver genes did not carry any potential driver mutations according to our strict criteria and were therefore excluded from subsequent analysis. In summary, 60 driver genes were identified (46 and 31 for MSS and MSI cases, respectively; Supplementary Tables 21 and 42).

### Evolutionary analysis

DPClust^53^ was run on all complete pipeline MSS samples with Battenberg data (*n* = 774) to identify clonal structure in each sample. The DPClust output was used in running MutationTimeR^36^ to annotate somatic mutations as early clonal, late clonal, subclonal or not available (NA) clonal (meaning unspecified clonality status). Samples with at least 256 early clonal and late clonal SBSs or 100 early clonal and late clonal IDs were retained and split into separate VCF files (*n* = 574 for SBS; *n* = 430 for ID). MSA^65^ was run on the resulting VCF files to identify the active mutational signatures in the early clonal and late clonal mutations. SBS signatures that were found to generate early clonal SBSs in fewer than 50 samples and also generated late clonal SBSs in fewer than 50 samples were excluded from the analysis. Similarly, ID signatures generating early clonal IDs in fewer than 20 samples and late clonal IDs in fewer than 20 samples were also excluded. Wilcoxon signed-rank tests were used to assess the differences in the relative activity of each signature between the early clonal and late clonal mutations. *P* values were adjusted across signatures using the Benjamini–Hochberg method^72^, and adjusted *P* values were reported as *q* values. This process was repeated with the same thresholds for SBSs and IDs to also assess the difference in the relative activity of each signature between clonal and subclonal mutations (*n* = 133 for SBS; *n* = 64 for ID). Due to the lower numbers, signatures that were found to generate clonal somatic mutations in fewer than ten samples and also generated subclonal somatic mutations in fewer than ten samples were excluded from the analysis.

### Motif analysis

MutaGene^73^ was used to find the number of mutations with the WAWW[T>N]W motif, previously associated with colibactin mutagenesis^7^, in each sample, regardless of the DNA strand. This value was then divided by the total number of W[T>N]W mutations per sample to identify the percentage of W[T>N]W mutations with the colibactin mutational motif.

### Microbiome analysis

To identify microbial reads that map to the pks island (*pks*), non-human reads were aligned to the IHE3034 genome (RefSeq assembly: GCF_000025745.1) using Bowtie2^74^. IHE3034 is a *pks E. coli* strain that contains the *pks* island with all 19 *clb* genes in the *clbA*–*clbS* gene cluster. Prior to alignment, poor quality reads were filtered using fastp^75^, and the remaining human reads were removed by excluding those that mapped to GRCh38, T2T-CHM13v2.0, and the 47 pangenomes^76^. A sample was considered *pks*^+^ if it had at least one read across at least 8 out of the 19 genes in the *clbA*–*clbS* gene cluster. Genome coverage circos plots were generated using reads per kilobase per million (RPKM) values and visualized with the circlize R package^77^.

### Regressions

To compare the mutation burden of different variant types, a linear regression of the mutation burden logarithm (base 10) was considered, using age, sex, tumour subsite, country and tumour purity as independent variables. For mutational signature-based analyses, signature attributions were dichotomized into presence and absence using confidence intervals, with presence defined as both lower and upper limits being positive and absence as the lower limit being zero (Supplementary Tables 16, 19, 20 and 43–46). If a signature was present in at least 70% of cases (SBS1, SBS5, SBS18, ID1, ID2, ID14 and CN2 for MSS cases; ID1, ID2, DBS_B_MSI, CN1 and SV_D for MSI cases), it was dichotomized into above and below the median of attributed mutation counts. The binary attributions served as dependent variables in logistic regressions. Regressions with variables presenting complete or quasi-complete separation^78^ were performed using Firth’s bias-reduced logistic regressions based on the logistf R package. To adjust for confounding factors, sex, age of diagnosis, tumour subsite, country and tumour purity were added as covariates in all regressions, serving as independent variables for the regressions. The tumour subsite variable was categorized as proximal colon (ICD-10-CM codes C18.0, C18.2, C18.3 and C18.4), distal colon (C18.5, C18.6 and C18.7) or rectum (C19 and C20), unless otherwise specified. One MSI tumour from an unspecified subsite was removed for the multivariable regression models in MSI cases. The age of diagnosis variable was generally considered as a numerical variable, or categorized into two (early-onset, <50 years old; and late-onset, ≥50 years) or five subgroups (0–39, 40−49, 50−59, 60–69, ≥70 years old), depending on the analysis performed, with specific indications in the corresponding figure legends. Similarly, regressions for driver mutations in cancer driver genes, hotspot driver mutations (present in at least ten cases), and pathogenic and likely pathogenic germline variants were done using the same logistic regression models but replacing signature by driver mutation prevalence across samples (Supplementary Tables 22–25 and 47−57).

Regressions with colorectal cancer incidence were performed as linear regressions with signature attributions with confidence intervals not consistent with zero as dependent variables, and ASRs of colorectal cancer (and independent ASR of colon and rectal cancer) obtained from the Global Cancer Observatory (GLOBOCAN)^1^, sex, age of diagnosis, tumour subsite and tumour purity as independent variables (Supplementary Tables 17 and 18). Regressions were performed on a sample basis.

Regressions with colibactin presence (based on genomic and/or microbiome-derived detection) were performed as linear regressions with age of diagnosis as the dependent variable, and sex, tumour subsite, country and tumour purity as independent variables.

### Additional statistical analyses

For regressions of signatures, driver mutations in cancer driver genes, and hotspot driver mutations, *P* values were adjusted for multiple comparisons based on the total number of decomposed reference mutational signatures considered per variant type (that is, 19 SBS, 7 DBS, 11 ID, 9 CN and 11 SV signatures for MSS cases; 18 SBS, 10 DBS, 2 ID, 4 CN and 4 SV for MSI cases), cancer genes (46 for MSS; 31 for MSI), or hotspot driver mutations (38 for MSS; 14 for MSI) using the Benjamini–Hochberg method^72^. For country enrichment analyses, the mutation burdens and binary attributions of mutational signatures were compared for each country against all others. Therefore, *P* values were also adjusted for multiple comparisons based on the total number of countries assessed (a total of 11 countries). Adjusted *P* values were reported as *q* values, with *q* values < 0.05 considered statistically significant. For age of diagnosis-based regressions of colibactin presence across tumour subsites, *P* values were adjusted and reported as *q* values based on the total number of tumour subsites assessed (a total of 3 tumour subsites). For the age of diagnosis trend enrichment analysis of signatures, *P* trends were reported, with *P* trends <0.05 considered statistically significant. For evidence of co-occurrence or mutual exclusivity of two signatures, two-sided Fisher’s exact tests were used, and *P* values were reported, with *P* < 0.05 considered statistically significant.

### Reporting summary

Further information on research design is available in the Nature Portfolio Reporting Summary linked to this article.

## Online content

Any methods, additional references, Nature Portfolio reporting summaries, source data, extended data, supplementary information, acknowledgements, peer review information; details of author contributions and competing interests; and statements of data and code availability are available at 10.1038/s41586-025-09025-8.

## Supplementary information

Supplementary Information This file contains a Supplementary Note, additional references and Supplementary Figs. 1–19.

Reporting Summary

Supplementary Tables This file contains Supplementary Tables 1–57.

## Data Availability

Whole-genome sequencing data, somatic mutations, and patient metadata are deposited in the European Genome-phenome Archive (EGA) associated with study EGAS00001003774. ASR values were extracted from IARC Cancer Today (https://gco.iarc.fr/today/en/dataviz). Classification of germline variants was obtained from ClinVar (https://www.ncbi.nlm.nih.gov/clinvar). Data from the rnaturalearthdata v1.0.0 (https://CRAN.R-project.org/package=rnaturalearthdata) were used to generate maps. All other data are provided in the accompanying Supplementary Tables.

## Code availability

All algorithms used for data analysis are publicly available with repositories noted within the respective method sections. The code used for regression analysis and figures is available at https://github.com/AlexandrovLab/Mutographs_CRC.
