# Supplementary material for: Geographic and age variations in mutational processes in colorectal cancer
Source: Nature. 2025 Apr 23;643(8070):230–40. doi: 10.1038/s41586-025-09025-8 (PMC12221974; doi:10.1038/s41586-025-09025-8)
Supplement: Supplementary file 1 — This file contains a Supplementary Note, additional references and Supplementary Figs. 1–19. [file 41586_2025_9025_MOESM1_ESM.pdf]

nature portfolio

https://doi.org/10.1038/s41586-025-09025-8

Supplementary information

Geographic and age variations in mutational processes in colorectal cancer

In the format provided by the authors and unedited

SUPPLEMENTARY INFORMATION

This file contains the following:

Supplementary Note

Supplementary Note References

Supplementary Figures

SUPPLEMENTARY NOTE

Identification of DNA repair-deficient cases

Microsatellite instability (MSI) was initially identified based on the observed mutational burdens and profiles (**Fig. 1*c***), as well as by identifying germline pathogenic mutations in the mismatch repair genes (*MLH1*, *MSH2*, *MSH6*, and *PMS2*) consistent with Lynch syndrome (**Supplementary Table 2**), classifying 153 colorectal cancers as MSI.

Apart from MSI, we also evaluated germline and somatic pathogenic mutations in specific genes (**Supplementary Table 3**), as well as the similarity of mutational profiles to previously known mutational signatures (**Supplementary Fig. 1-3**) to detect cases affected by other DNA repair deficiencies. This included the observation of specific mutations and signatures associated with defects in polymerase proofreading (*POLE* mutations and COSMICv3.4 SBS10a, SBS10b, and SBS28 signatures[1-3]; *POLD1* mutations and SBS10c[4]), homologous recombination repair (*BRCA1* and *BRCA2* mutations and SBS3 and ID6[5,6]); and base excision repair (*MUTYH* mutations and SBS36[7,8]; *NTHL1* mutations and SBS30[2,9]; *OGG1* mutations and Signal SBS108[10,11]). In addition, the Classifier of Homologous Recombination Deficiency (CHORD)[12] was run to refine the identification of homologous recombination repair deficient cases (**Supplementary Table 4**). In total, 10 cases were identified as harboring *POLE* mutations (with signatures associated with *POLE* mutations contributing on average 82.5% of mutations across all *POLE* mutated cases), 3 as harboring *POLD1* mutations (with *POLD1*-associated signatures contributing on average 61.1% of mutations), 7 as harboring homologous recombination deficiency (HRD; with HRD-associated signatures contributing on average 44.7% of indels), 2 as harboring *NTHL1* mutations (with *NTHL1*-associated signatures contributing on average 48.4% of mutations), 1 as harboring a

MUTYH mutation (with *MUTYH*-associated signatures contributing on average 83.2% of mutations), and 1 as harboring an *OGG1* mutation (with *OGG1*-associated signatures contributing on average 56.4% of mutations).

MSI tumors were enriched in the proximal colon (OR=12.2, p=3.8×10^{-27}) and more common in early-onset patients (OR=2.6, p=0.001), although this latter association was not observed after excluding cases attributed to Lynch syndrome (p>0.05). Similar to MSI, homologous recombination repair deficient cases were also predominantly found in the proximal colon (OR=7.7, p=0.009), whereas no other DNA repair deficiency was significantly enriched early-onset or late-onset colorectal cancer patients (p>0.05).

Validation of microsatellite instability status

The MSI status for 131 of the 153 cases initially classified as MSI, where DNA was available post whole-genome sequencing (**Supplementary Table 28**), was independently validated using droplet digital PCR (ddPCR; **Methods**). Of these, 130 cases (99.2%) identified as MSI using mutational burdens and profiles were confirmed by ddPCR (**Supplementary Table 28**). Interestingly, the one case classified as microsatellite stable (MSS) by ddPCR showed loss of *PMS2* by immunohistochemistry. Although no germline or somatic mutations were found in known mismatch repair genes, this tumor displayed a mutational profile compatible with the presence of MSI (**Supplementary Fig. 17**) and was therefore labeled as MSI for subsequent analyses.

Repertoire of mutational signatures in MSI colorectal cancers

De novo extraction detected 15 single base substitution (SBS), 3 small insertion and deletion (ID), 6 double base substitution (DBS), 3 copy number (CN), and 2 structural variant (SV) mutational signatures in the 153 MSI colorectal tumors (**Supplementary Tables 29-33**). Upon decomposition to known COSMICv3.4 signatures, the MSI cohort predominantly displayed signatures associated with mismatch repair deficiencies. Specifically, the analysis identified all MSI-associated SBS, DBS, and CN signatures[1,3,5,13], including SBS6, SBS14, SBS15, SBS20, SBS21, SBS26, SBS44, DBS7, DBS10, and CN25 (**Supplementary Tables 36-41**). Notably, three novel SBS signatures (SBS_I_MSI, SBS_N_MSI, and SBS_O_MSI) and one novel DBS signature (DBS_B_MSI) were also identified (**Supplementary Fig. 15*a-c***). Signature DBS_B_MSI was found in 114 of the 153 MSI colorectal tumors (74.5%), being the most prevalent DBS signature in 101 of these cases (**Supplementary Table 39**). In contrast, signatures SBS_I_MSI, SBS_N_MSI, and SBS_O_MSI were present in a small subset (<10% of all samples) of the MSI colorectal tumors, where they contributed many thousands of somatic mutations, being the most prevalent SBS signatures in a total of five cases (three cases for SBS_I_MSI, one for SBS_N_MSI, and one for SBS_O_MSI; **Supplementary Fig. 15*d***; **Supplementary Table 37**). SBS_M_MSI was not found in COSMICv3.4, but the signature was previously observed in a handful of breast, central nervous system, colorectal, and prostate tumors from Dutch and British patients, contributing a high amount of mutations and being associated with mismatch repair deficiency[10]. Similarly, in our cohort, SBS_M_MSI was only found present in 13 of the 153 MSI cases (8.5%), being the most prevalent signature in only one of them (**Supplementary Fig. 15*e***; **Supplementary Table 37**). Lastly, an MSI-associated artifactual signature DBS14, caused by the difficulty in aligning regions of long T or A repeats[10,13], was found in 7 of the 153 MSI colorectal cancers (4.6%).

In addition to MSI-associated signatures, our analysis identified other mutational signatures operative within the MSI cohort, including clock-like signatures SBS1, SBS5, ID1, and ID2[3,5], reactive oxygen species-linked signature SBS18[3], acetaldehyde-linked signature DBS2[5], ploidy-linked signatures CN1, CN2, and CN9[14], and homologous recombination deficiency associated signature SV3[2,13]. As previously reported[15], the number of mutations contributed by clock-like signatures was elevated in the MSI cases when compared to the MSS cases, with ID2 showing the highest elevation (median fold-change=273.7, p=9.9×10^{-86}). Additionally, mutational signatures with unknown etiology were also detected within the MSI cohort, including SBS17a/b, SBS34, SBS93, DBS4, DBS8, DBS15, DBS16, DBS18, SV3, SV5, SV7, and SV_D, where SV_D was previously extracted in the MSS colorectal cancer cohort (**Methods**).

Cancer driver genes in MSI colorectal cancers

Consensus driver gene identification using the Intogen framework[16] identified a total of 31 cancer genes under positive selection in the MSI cases, 17 of them shared with the MSS cohort (**Supplementary Table 42**). Twenty-nine of these driver genes were previously reported in colorectal cancer[16,17] and two in other cancer types (*DDX3X* and *ACSL6*)[16]. We also identified *CDKN1B*, *TCF3*, and *TOP1* for the first time as driver genes for MSI colorectal cancer, after being reported recently as colorectal cancer drivers in UK patients exclusively for the MSS molecular subgroup[17].

Geographic and age-related variations in mutational signatures and driver genes in MSI colorectal cancers

Despite exhibiting a significantly higher number of substitutions than MSS tumors (95,426 vs. 12,054; FC=7.92; $p=1.6\times10^{-85}$), MSI colorectal cancers display a lower diversity of mutational signatures due to the dominance of mismatch repair deficiency-associated signatures, which account for the majority of somatic mutations. Furthermore, limited geographic or age-related differences were observed in MSI cases, likely attributed to the smaller sample size and the predominance of mutations driven by defective DNA repair mechanisms. In particular, only mutational signature ID1 was found at significantly lower levels in the Czech Republic (OR=0.04, q=0.025), whereas no other statistically significant differences across countries were observed for either the prevalence of mutational signatures (q>0.05; **Supplementary Table 43**), or driver mutations affecting detected cancer driver genes (q>0.05; **Supplementary Table 47**), or hotspot driver mutations (q>0.05; **Supplementary Table 48**) across MSI cases, after adjusting by the effect of age, sex, tumor subsite, and tumor purity. Signature CN25, previously associated with mismatch repair deficiency[13], was found enriched in MSI patients older than 50 years (OR=20.0, q=0.012), while no other MSI signatures were found to be associated with the age of tumor onset (q>0.05; **Supplementary Table 44**). In addition, driver mutations in *APC* (OR=10.5, $q=3.0\times10^{-4}$) and *PIK3CA* (OR=8.1, q=0.004) were enriched in early-onset MSI patients, while *BRAF* (OR=25.5, q=0.002) and *RNF43* (OR=4.3, q=0.045) were enriched in late-onset MSI patients (**Supplementary Table 49**). In particular, the enrichment for *BRAF* mutations was driven by the p.V600E *BRAF* hotspot mutation (OR=21.9, q=0.005; **Supplementary Table 50**), which accounted for 55 out of the total 58 *BRAF* mutations, all of them present in late-onset MSI patients.

After excluding the 31 Lynch syndrome MSI cases, no additional associations were observed for mutational signatures, driver mutations, geographic locations, or ages of onset (**Supplementary Tables 45-46** and **51-54**; q>0.05), with ID1 burden still decreased in the Czech Republic (OR=0.03, q=0.020; **Supplementary Table 45**) and *APC* (OR=13.5, q=0.009; **Supplementary Table 53**) enriched in early-onset MSI cases, and *BRAF* (OR=20.1, q=0.029; **Supplementary Table 53**) and the p.V600E *BRAF* hotspot mutation (OR=17.9, q=0.042; **Supplementary Table 54**) enriched in late-onset MSI cases.

Germline variants in MSS and MSI colorectal cancers

To access the impact of pathogenic or likely pathogenic germline variants, we access the ClinVar status of germline variants in 292 colorectal cancer predisposition syndromes genes[18], homologous recombination genes (HR)[19], and DNA damage repair genes (DDR)[20]. We identified a total of 127 pathogenic or likely pathogenic germline variants in 41 of the genes analyzed **(Supplementary Table 55**). The MSS cases harbored 78 variants in 75 cases, including one variant in a gene related to Cowden syndrome (*SEC23B*), two variants on mismatch repair genes related to Lynch syndrome (*MLH1* and *MSH6*), 52 variants on DDR-genes, 23 on HR-genes. None of these variants showed enrichment in early-onset MSS cases (**Supplementary Fig. 18*a***; **Supplementary Table 56**). In the MSI cases, in addition to the 31 variants in DNA mismatch repair genes associated with Lynch syndrome, we also identified 10 pathogenic or likely pathogenic variants on DDR-genes, *BRCA1*, and *SEC23B* (**Supplementary Table 55**). Cases harboring pathogenic or likely pathogenic variants in DNA mismatch repair genes consistent with Lynch syndrome showed enrichment in early-onset patients (OR=4.8, q=0.010; **Supplementary Fig. 18*b***; **Supplementary Table 57**).

Microbiome analysis of signatures enriched in early-onset MSS colorectal cancers

We selected genera with a minimum abundance of 1,000 reads across all samples and filtered out those with low overall variance in the dataset. This process resulted in 91 genera for association analysis with mutational signatures. To ensure an unbiased analysis, we stratified the mutational signatures into binary classes and applied a binomial logistic regression model. For each mutational signature, we conducted a separate regression model for each of the 91 genera. We included the genus as a covariate alongside all other covariates previously included in our regression models (age of diagnosis, sex, tumor subsite, country, and tumor purity). In addition, we also included as a covariate the total number of reads, as a proxy for sequencing depth, considering its potential impact on the microbiome analysis. After applying false discovery rate (FDR) p-value correction, we did not identify any significant association between specific genera and SBS89, SBS_M, or ID14.

As an alternative analysis, to address potential multicollinearity among the genera and reduce the number of genus-specific models, we also performed a hierarchical clustering using the Bray-Curtis distance metric. This approach grouped the 91 genera identified in the initial analysis into 50 clusters, using a distance threshold of 0.70 (**Supplementary Fig. 19**). For each mutational signature, we conducted a separate regression model for each of the 50 clusters, replacing the individual genera used previously. Each model included the cluster as a covariate alongside the same base-level covariates specified previously. By incorporating clusters instead of individual genera, we reduced the dimensionality of the data and accounted for correlated features. This clustering-based approach allowed us to test associations between microbial community structures and mutational signatures. However, even with this adjustment, we did not find significant

associations for SBS89, SBS_M, or ID14. Further exploration of microbial data in future studies, potentially integrating additional cases harboring these signatures, more detailed metadata, or functional microbial pathways, could provide insights into the underlying causes of SBS89, SBS_M, and ID14, and their effect on early colorectal mutagenesis.

SUPPLEMENTARY NOTE REFERENCES

1 Alexandrov, L. B. *et al.* Clock-like mutational processes in human somatic cells. *Nat Genet* **47**, 1402-1407 (2015). https://doi.org/10.1038/ng.3441

2 Nik-Zainal, S. *et al.* Landscape of somatic mutations in 560 breast cancer whole-genome sequences. *Nature* **534**, 47-54 (2016). https://doi.org/10.1038/nature17676

3 Alexandrov, L. B. *et al.* Signatures of mutational processes in human cancer. *Nature* **500**, 415-421 (2013). https://doi.org/10.1038/nature12477

4 Robinson, P. S. *et al.* Increased somatic mutation burdens in normal human cells due to defective DNA polymerases. *Nature Genetics* **53**, 1434-1442 (2021). https://doi.org/10.1038/s41588-021-00930-y

5 Alexandrov, L. B. *et al.* The repertoire of mutational signatures in human cancer. *Nature* **578**, 94-101 (2020). https://doi.org/10.1038/s41586-020-1943-3

6 Nik-Zainal, S. *et al.* Mutational processes molding the genomes of 21 breast cancers. *Cell* **149**, 979-993 (2012). https://doi.org/10.1016/j.cell.2012.04.024

7 Pilati, C. *et al.* Mutational signature analysis identifies MUTYH deficiency in colorectal cancers and adrenocortical carcinomas. *J Pathol* **242**, 10-15 (2017). https://doi.org/10.1002/path.4880

8 Viel, A. *et al.* A Specific Mutational Signature Associated with DNA 8-Oxoguanine Persistence in MUTYH-defective Colorectal Cancer. *EBioMedicine* **20**, 39-49 (2017). https://doi.org/10.1016/j.ebiom.2017.04.022

9 Grolleman, J. E. *et al.* Mutational Signature Analysis Reveals NTHL1 Deficiency to Cause a Multi-tumor Phenotype. *Cancer Cell* **35**, 256-266 e255 (2019). https://doi.org/10.1016/j.ccell.2018.12.011

10 Degasperi, A. *et al.* Substitution mutational signatures in whole-genome-sequenced cancers in the UK population. *Science* **376**, abl9283 (2022). https://doi.org/10.1126/science.abl9283

11 Zou, X. *et al.* A systematic CRISPR screen defines mutational mechanisms underpinning signatures caused by replication errors and endogenous DNA damage. *Nature Cancer* **2**, 643-657 (2021). https://doi.org/10.1038/s43018-021-00200-0

12 Nguyen, L., W. M. Martens, J., Van Hoeck, A. & Cuppen, E. Pan-cancer landscape of homologous recombination deficiency. *Nature Communications* **11**, 5584 (2020). https://doi.org/10.1038/s41467-020-19406-4

13 Everall, A. *et al.* Comprehensive repertoire of the chromosomal alteration and mutational signatures across 16 cancer types from 10,983 cancer patients. *medRxiv*, 2023.2006.2007.23290970 (2023). https://doi.org/10.1101/2023.06.07.23290970

14 Steele, C. D. *et al.* Signatures of copy number alterations in human cancer. *Nature* **606**, 984-991 (2022). https://doi.org/10.1038/s41586-022-04738-6

15 Díaz-Gay, M. & Alexandrov, L. B. in *Advances in Cancer Research* Vol. 151 (eds Franklin G. Berger & C. Richard Boland) 385-424 (Academic Press, 2021).

16 Martinez-Jimenez, F. *et al.* A compendium of mutational cancer driver genes. *Nat Rev Cancer* **20**, 555-572 (2020). https://doi.org/10.1038/s41568-020-0290-x

17 Cornish, A. J. *et al.* The genomic landscape of 2,023 colorectal cancers. *Nature* (2024). https://doi.org/10.1038/s41586-024-07747-9

18 Board, W. C. o. T. E. *WHO classification of Tumours: Digestive System Tumours*. 5th edn, (World Health Organization, 2019).

19 Riaz, N. *et al.* Pan-cancer analysis of bi-allelic alterations in homologous recombination DNA repair genes. *Nature Communications* **8**, 857 (2017). https://doi.org/10.1038/s41467-017-00921-w
20 Knijnenburg, T. A. *et al.* Genomic and Molecular Landscape of DNA Damage Repair Deficiency across The Cancer Genome Atlas. *Cell Reports* **23**, 239-254.e236 (2018). https://doi.org/10.1016/j.celrep.2018.03.076

SUPPLEMENTARY FIGURES

Supplementary Figure 1

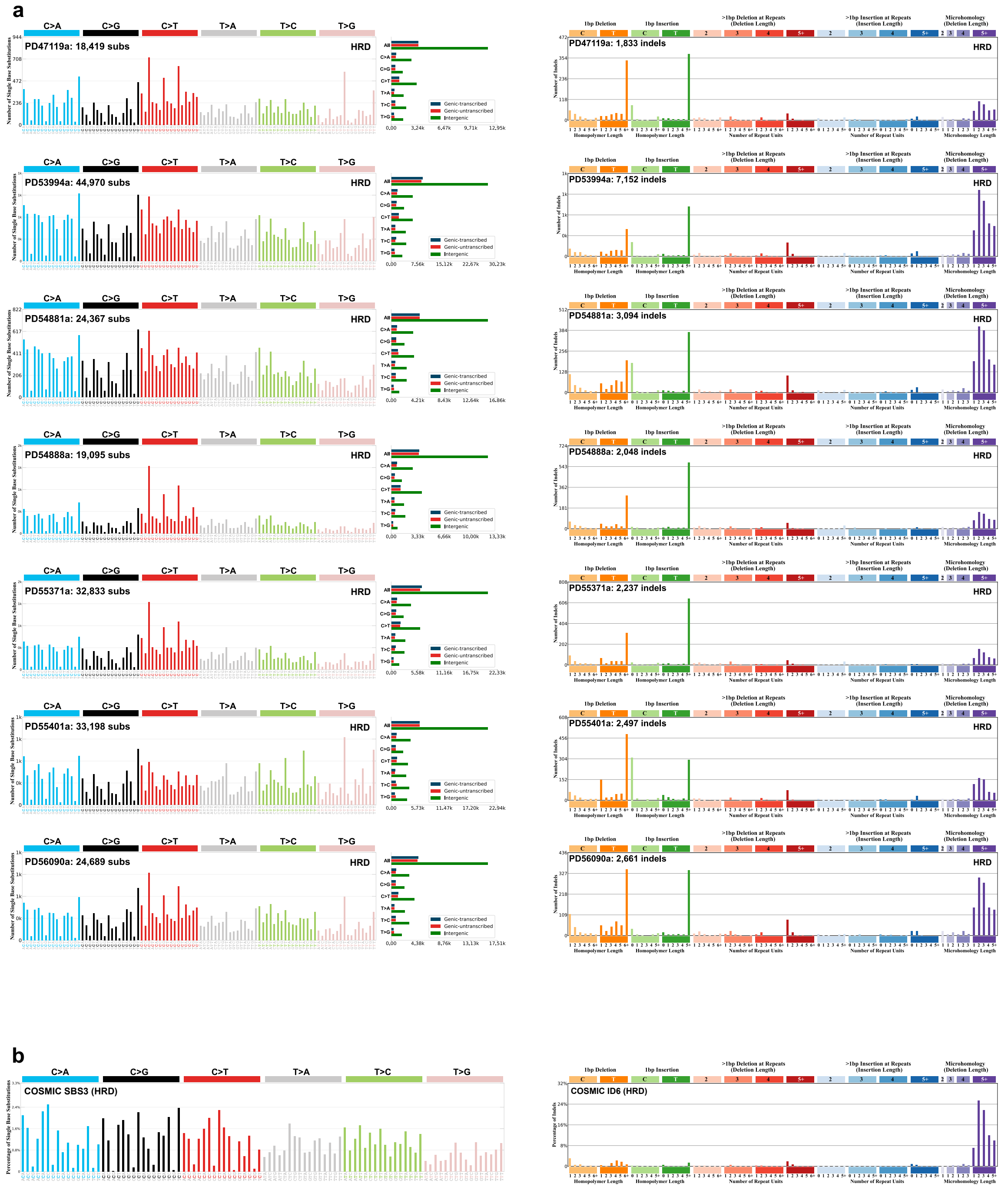

Supplementary Fig. 1. Mutational profiles of homologous recombination deficient colorectal cancers. a, Mutational profiles of individual samples identified as homologous recombination

deficient for single base substitutions (SBS-288 mutational context) and small insertions and deletions (ID-83 mutational context). **b**, Mutational signatures previously associated with homologous recombination deficiency in COSMICv3.4 (SBS3 and ID6).

Supplementary Figure 2

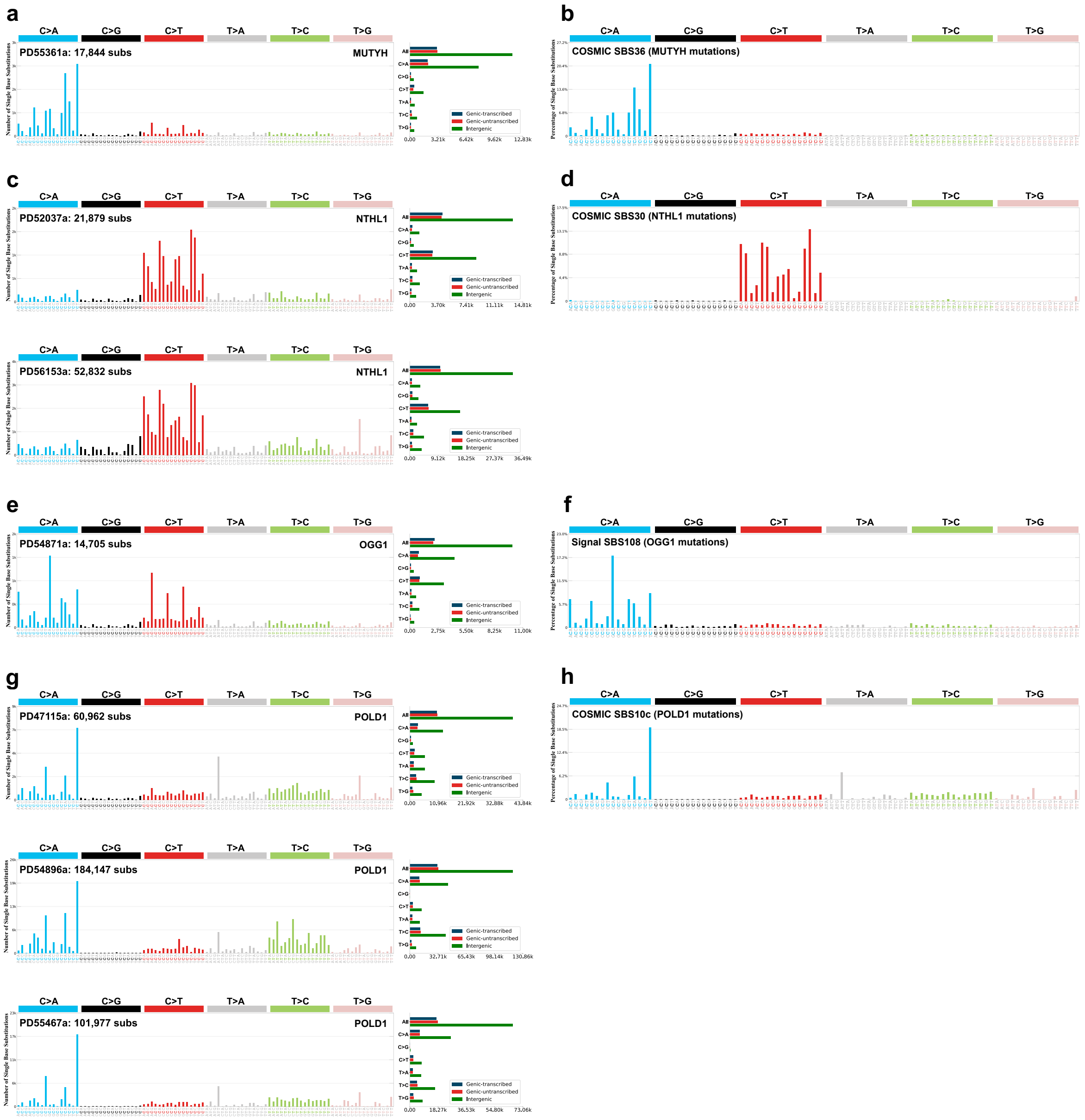

Supplementary Fig. 2. Mutational profiles of base excision repair deficient and *POLD1* mutated colorectal cancers. a, Mutational profile of an individual sample identified as base excision repair deficient due to mutations in *MUTYH* for single base substitutions (SBS-288 mutational context). **b**, Mutational signature previously associated with base excision repair deficiency due to mutations in *MUTYH* in COSMICv3.4 (SBS36). **c**, Mutational profiles of

individual samples identified as base excision repair deficient due to mutations in *NTHL1* for single base substitutions (SBS-288 mutational context). **d**, Mutational signature previously associated with base excision repair deficiency due to mutations in *NTHL1* in COSMICv3.4 (SBS30). **e**, Mutational profile of an individual sample identified as base excision repair deficient due to mutations in *OGG1* for single base substitutions (SBS-288 mutational context). **f**, Mutational signature previously associated with base excision repair deficiency due to mutations in *OGG1* (Signal signature SBS108). **g**, Mutational profiles of individual samples harboring mutations in *POLD1* for single base substitutions (SBS-288 mutational context). **h**, Mutational signature previously associated with mutations in *POLD1* in COSMICv3.4 (SBS10c).

Supplementary Figure 3

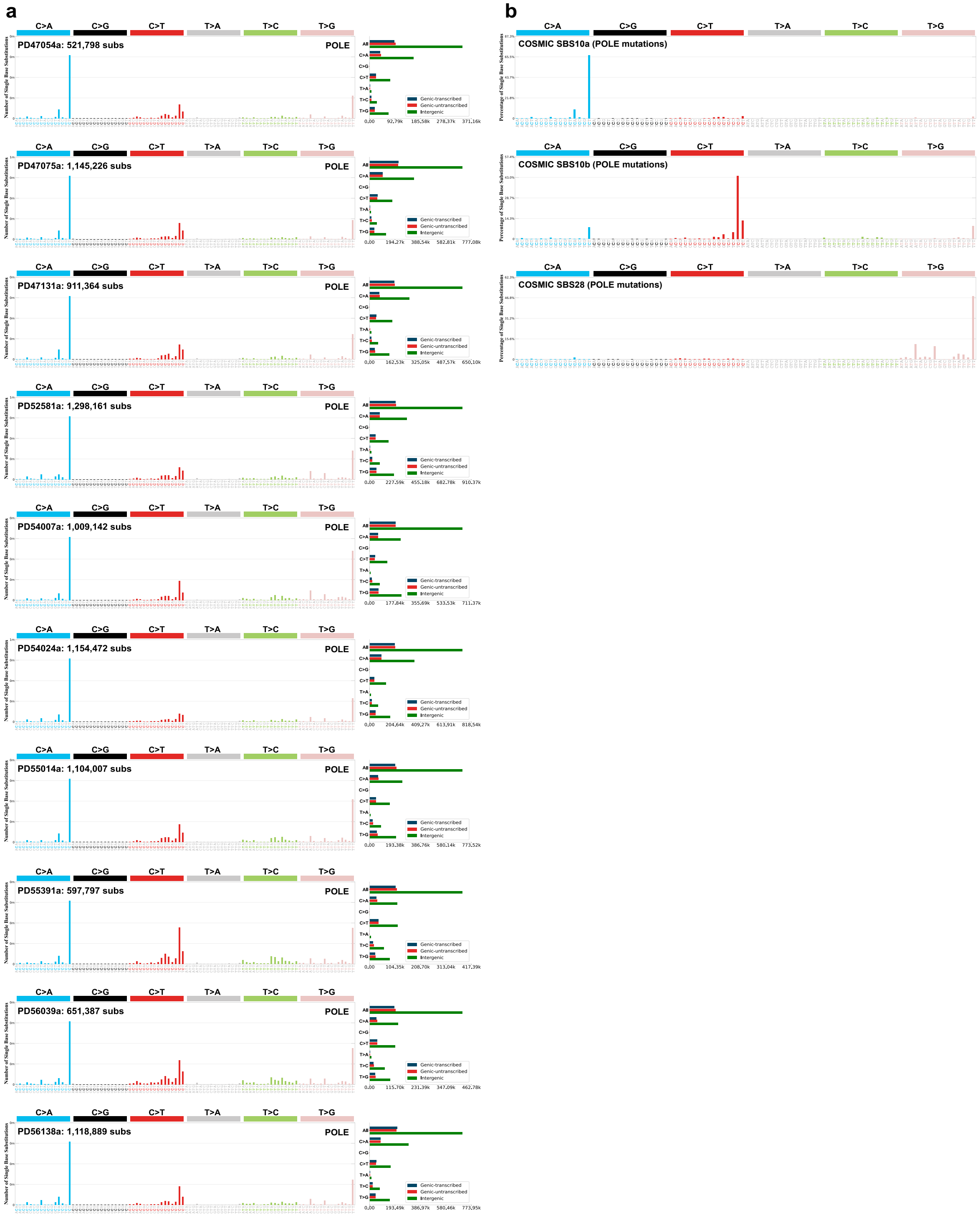

Supplementary Fig. 3. Mutational profiles of *POLE* mutated colorectal cancers. a, Mutational profiles of individual samples harboring mutations in *POLE* for single base substitutions (SBS-

288 mutational context). **b**, Mutational signatures previously associated with mutations in *POLE* in COSMICv3.4 (SBS10a, SBS10b, and SBS28).

Supplementary Figure 4

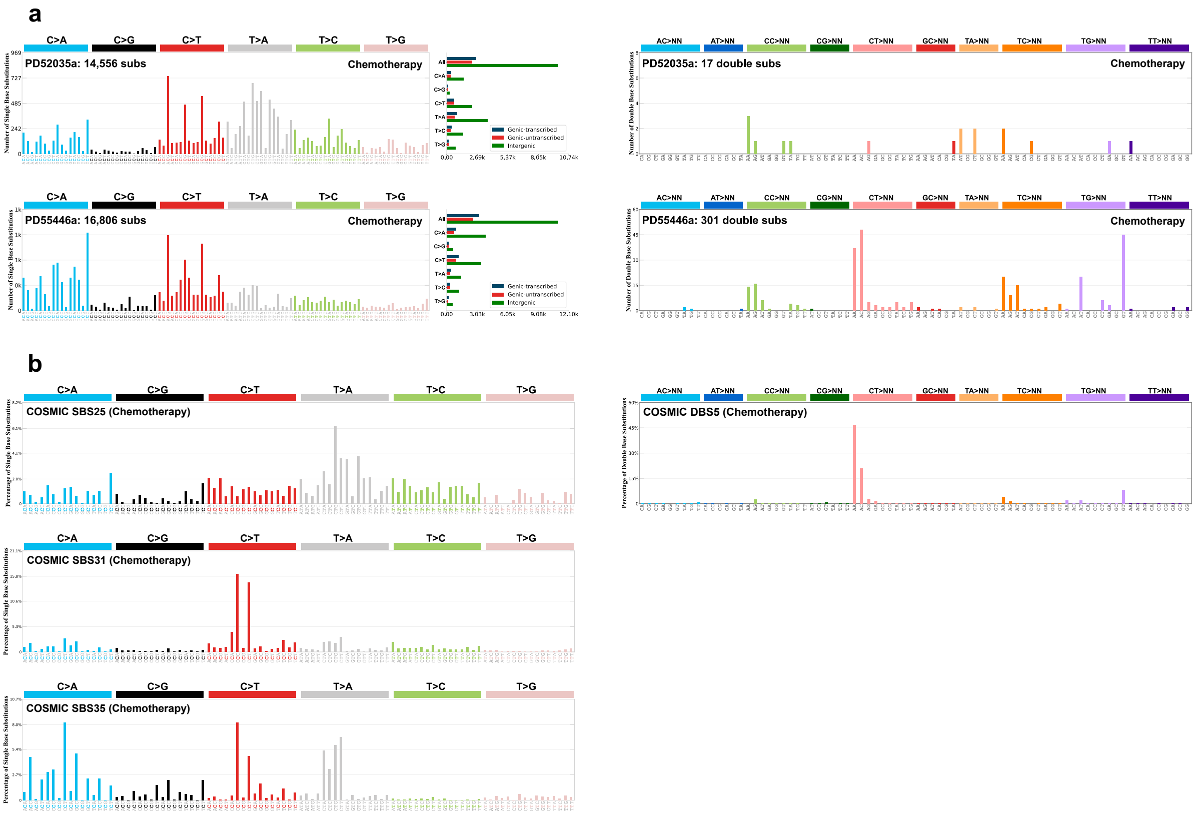

Supplementary Fig. 4. Mutational profiles of cases treated with chemotherapy for prior cancers. a, Mutational profiles of individual samples treated with chemotherapy for prior cancers for single base substitutions (SBS-288 mutational context) and doublet base substitutions (DBS-78 mutational context). **b**, Mutational signatures previously associated with chemotherapy in COSMICv3.4 (SBS25, SBS31, SBS35, and DBS5).

Supplementary Figure 5

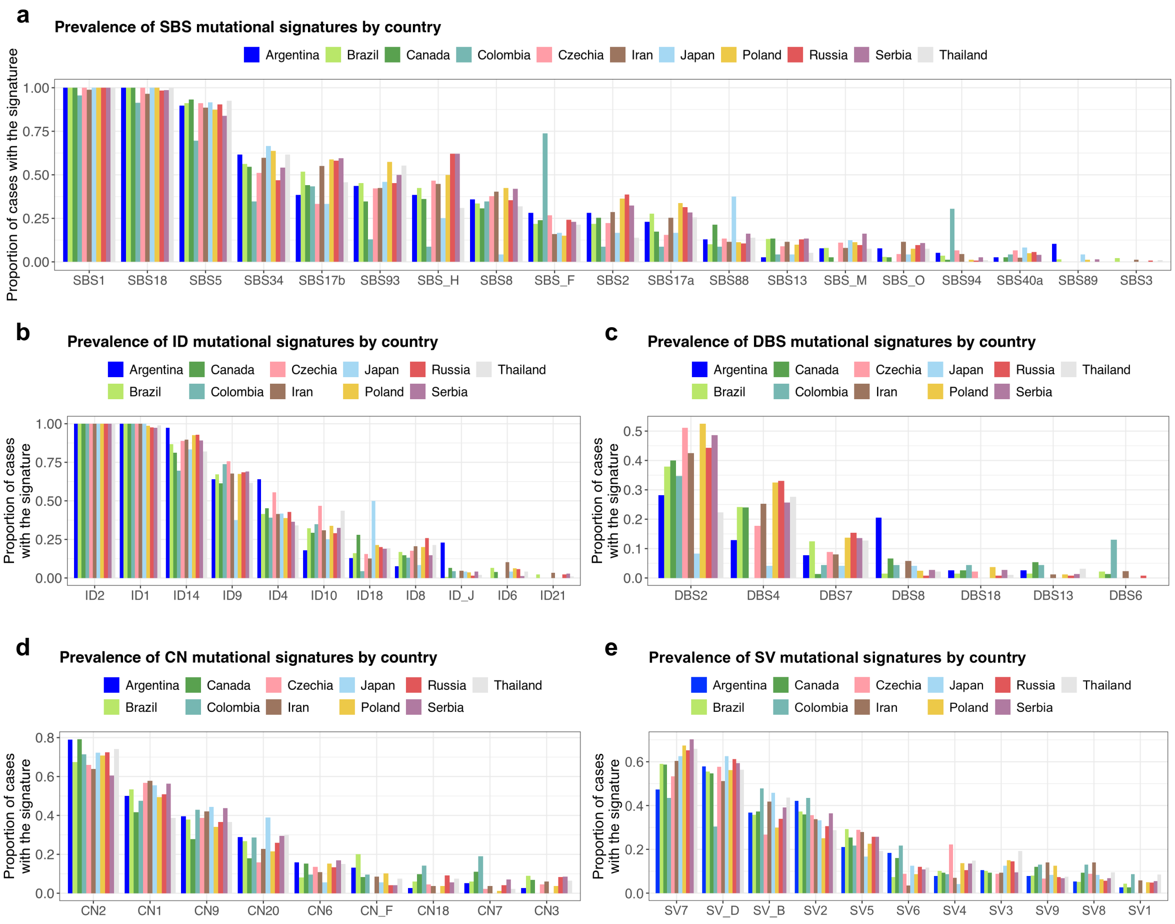

Supplementary Fig. 5. Prevalence of mutational signatures in microsatellite stable colorectal cancers by country. a-e, SBS (**a**), ID (**b**), DBS (**c**), CN (**d**), and SV (**e**) signatures.

Supplementary Figure 6

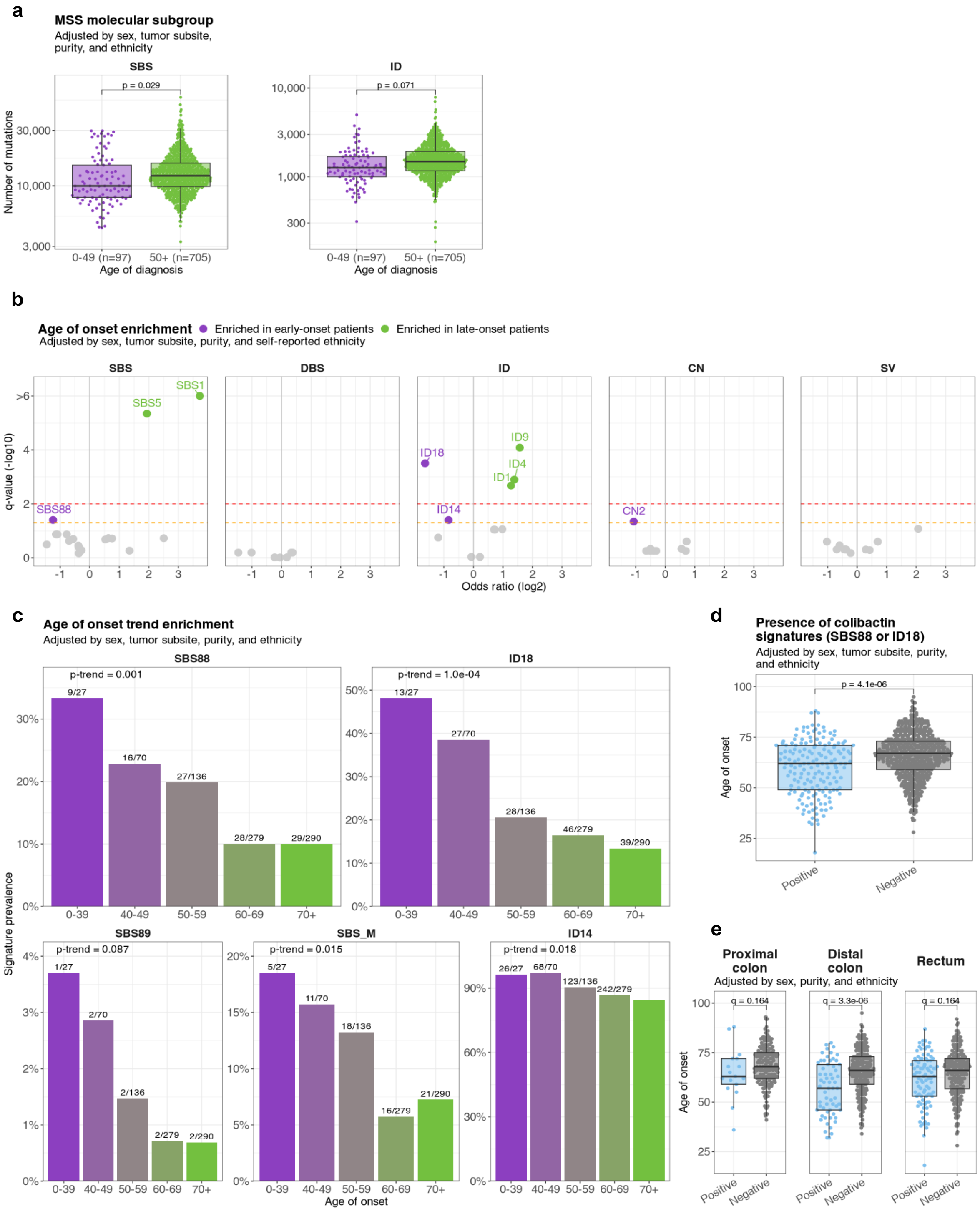

Supplementary Fig. 6. Main age association analyses adjusted by self-reported ethnicity instead of country of origin. a-e, Replicates of Fig. 1d (**a**), Fig.3a (**b**), and Fig. 3c-e (**c-e**).

Supplementary Figure 7

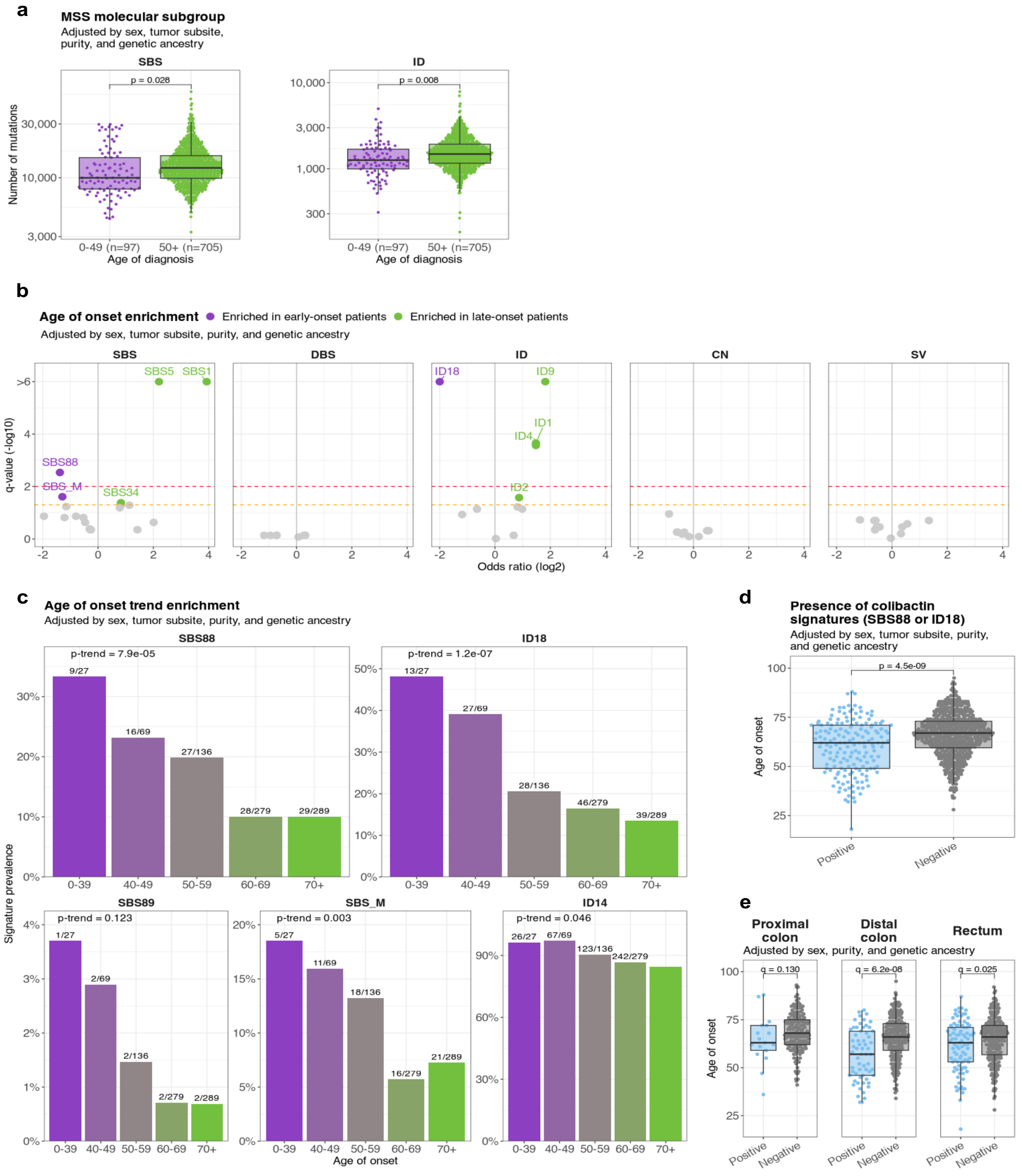

Supplementary Fig. 7. Main age association analyses adjusted by the first five principal components of the genetic ancestry analysis instead of country of origin. a-e, Replicates of Fig. 1d (**a**), Fig.3a (**b**), and Fig. 3c-e (**c-e**).

Supplementary Figure 8

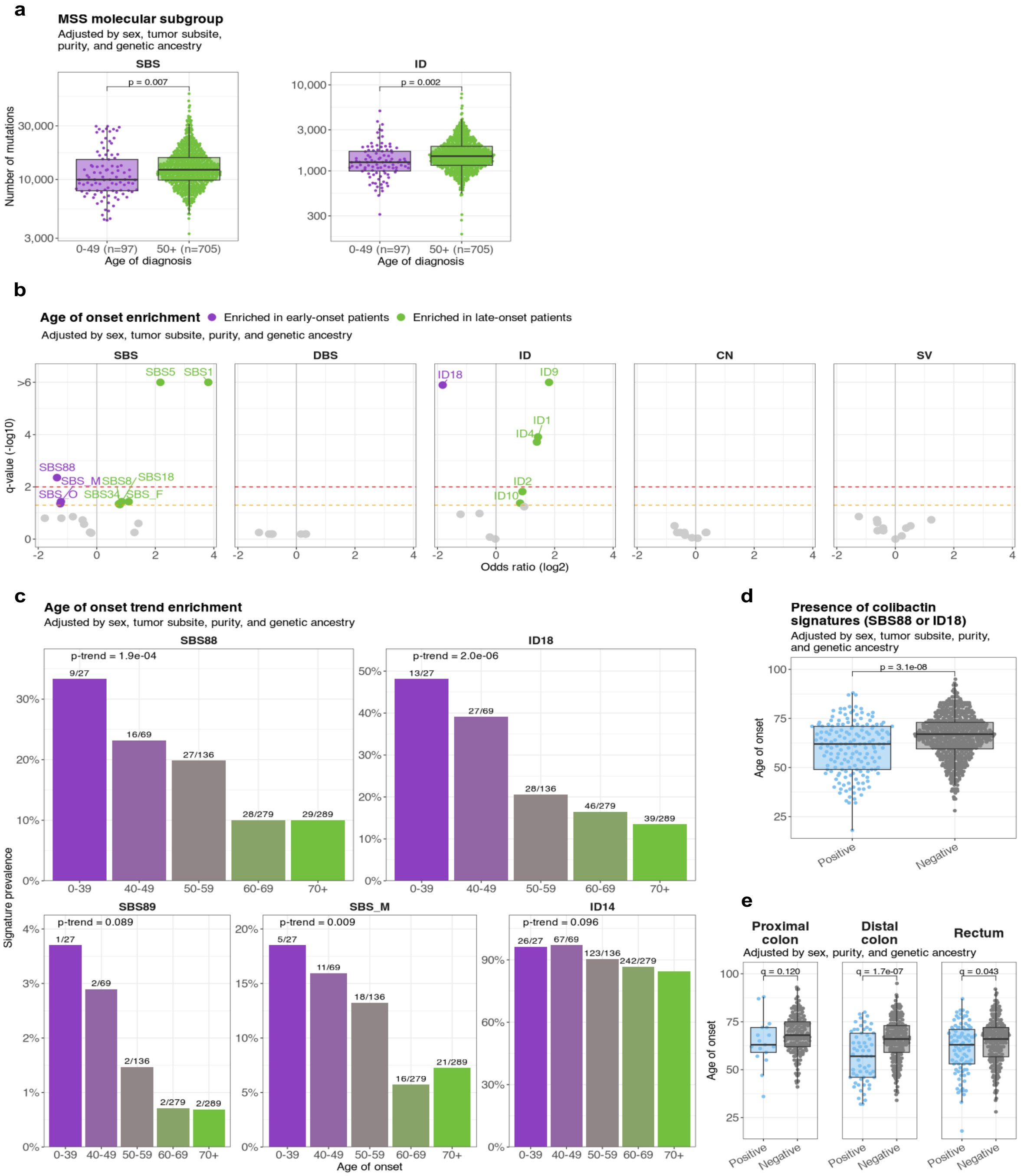

Supplementary Fig. 8. Main age association analyses adjusted by genetic ancestry groups (ADMIX, AFR, EAS, EUR) instead of country of origin. a-e, Replicates of Fig. 1d (**a**), Fig.3a (**b**), and Fig. 3c-e (**c-e**).

Supplementary Figure 9

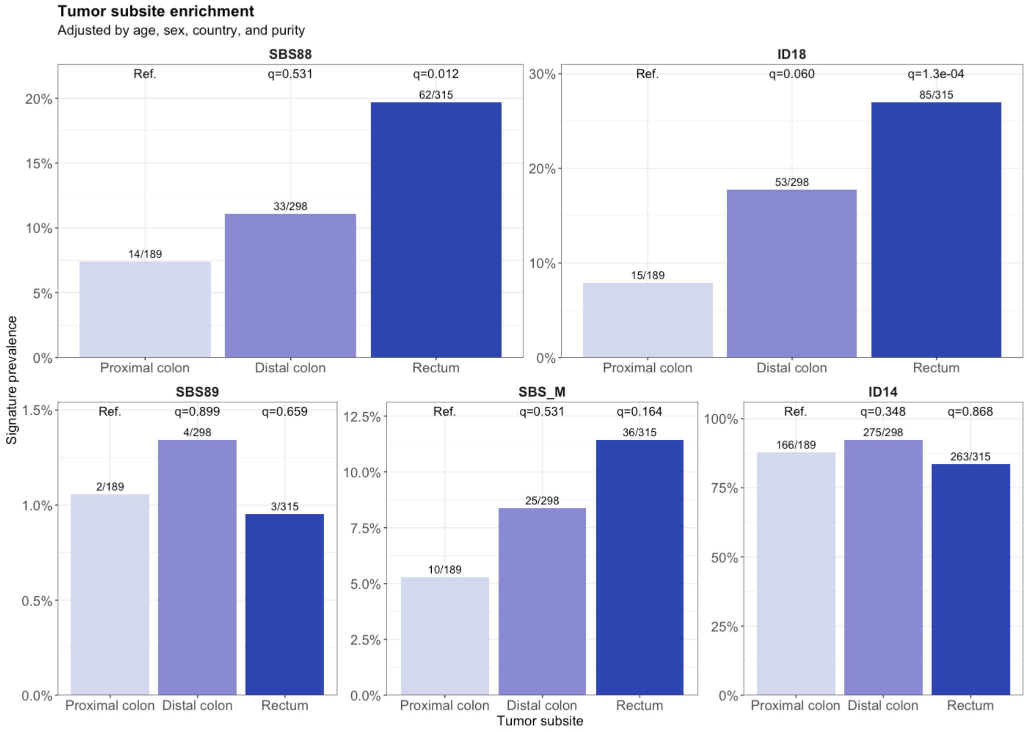

Supplementary Fig. 9. Variation of mutational signatures associated with earlier age of onset with tumor subsite in microsatellite stable colorectal cancers.

Supplementary Figure 10

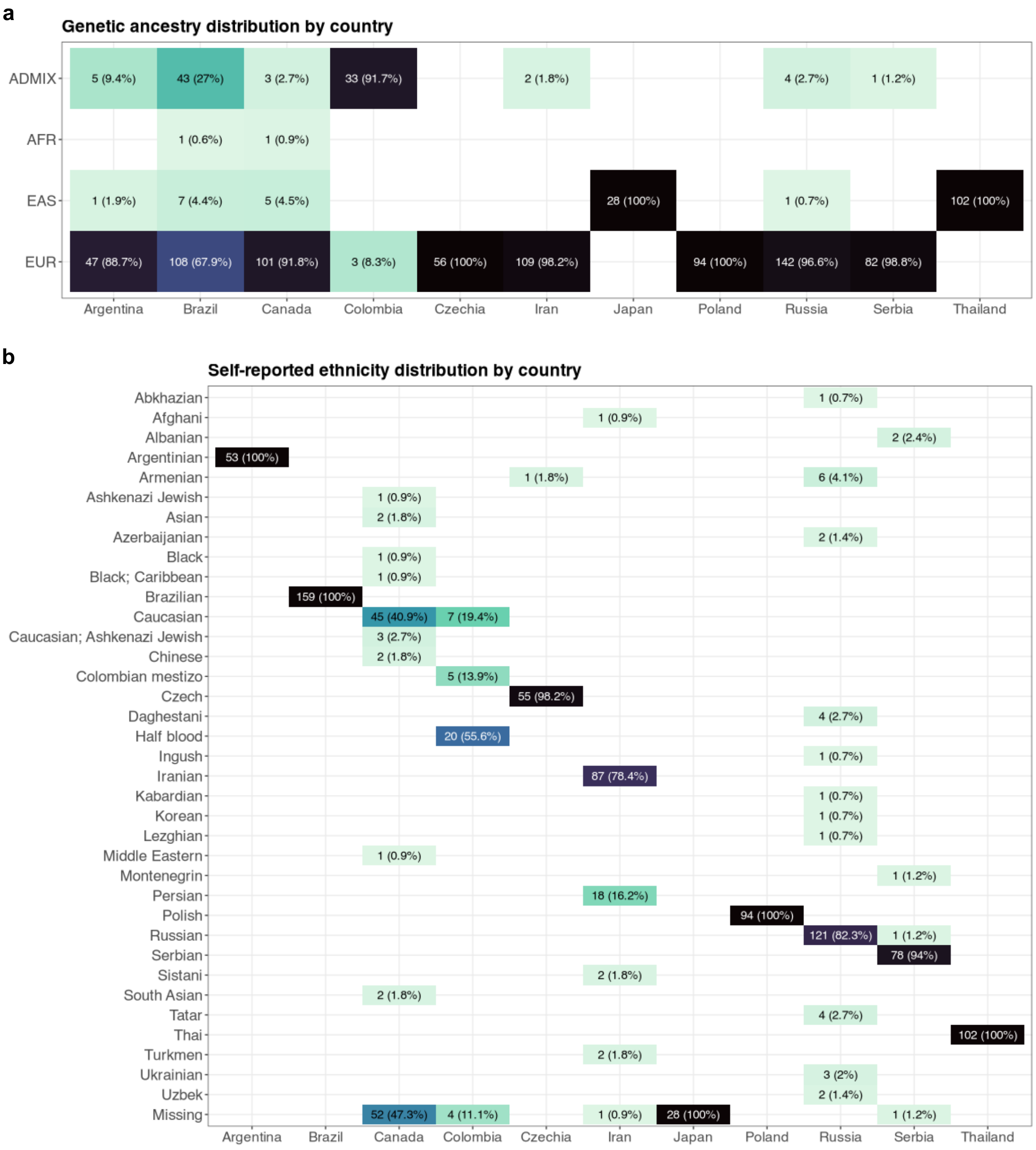

Supplementary Fig. 10. Genetic ancestry (a) and self-reported ethnicity (b) distribution by country.

Supplementary Figure 11

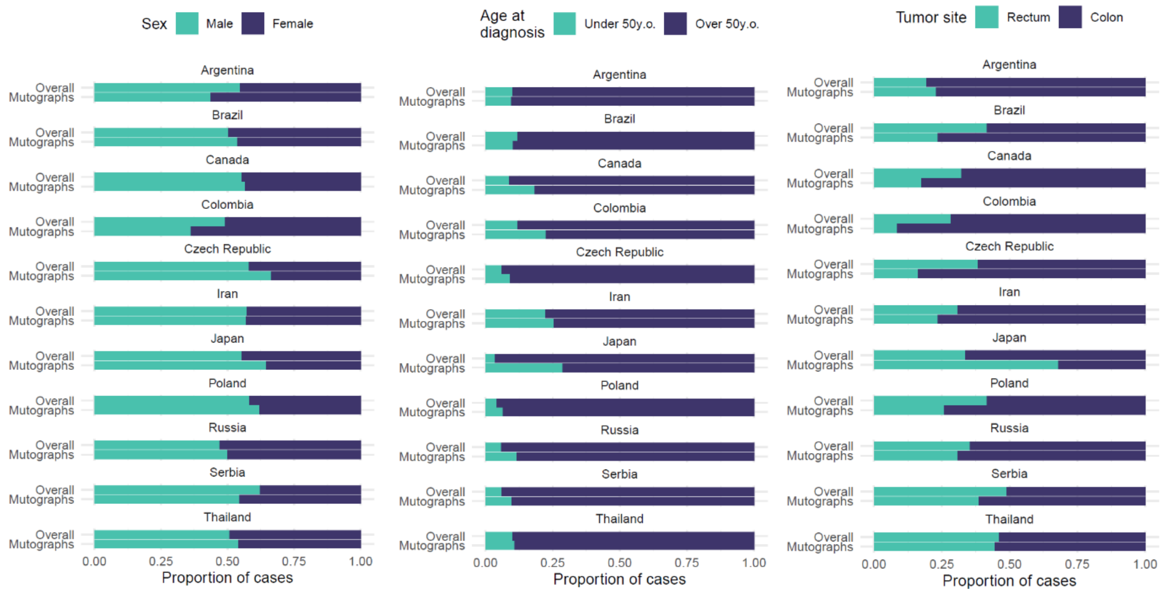

Supplementary Fig. 11. Comparison of clinicopathological characteristics at baseline by country, comparing Mutographs samples vs. GLOBOCAN-based expectations.

Supplementary Figure 12

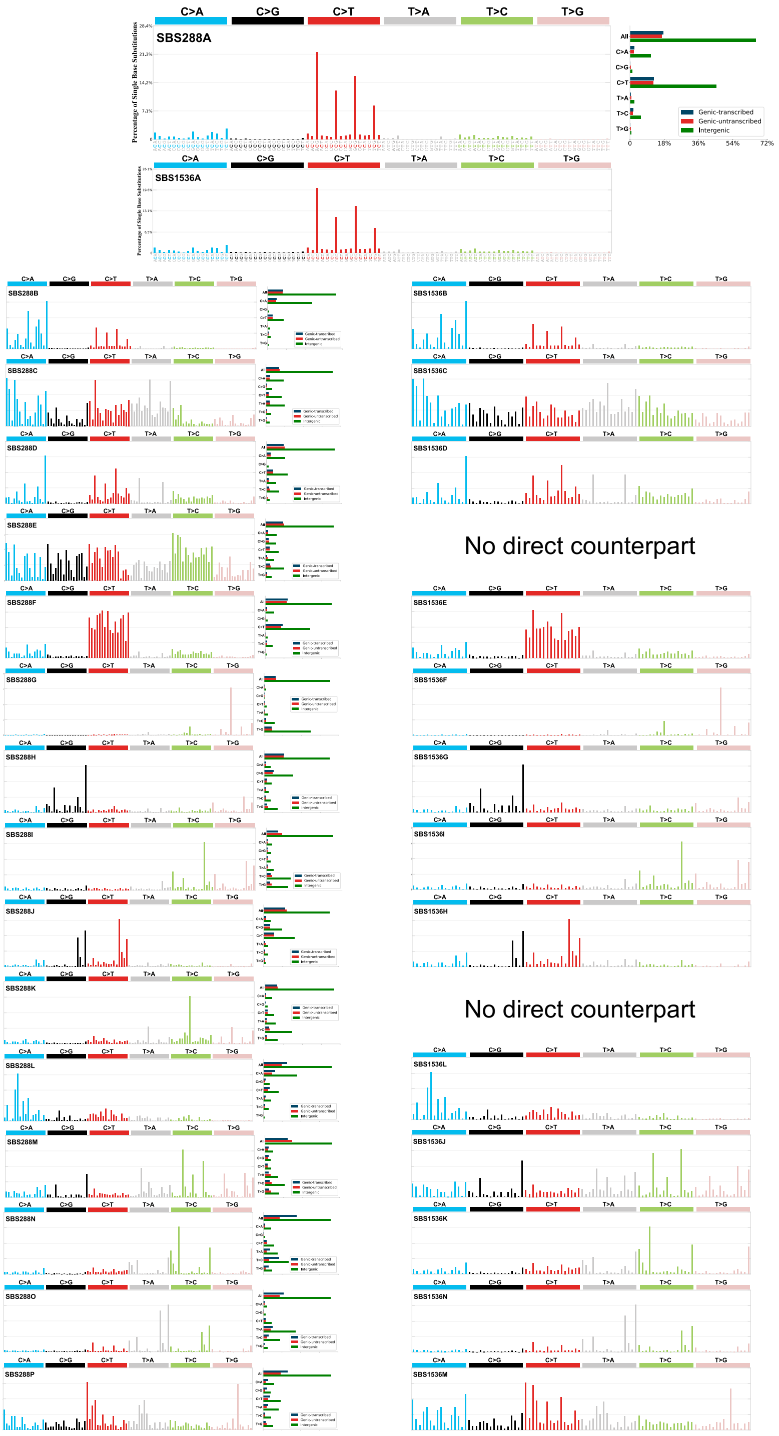

Supplementary Fig. 12. Single base substitution mutational signatures extracted by SigProfilerExtractor using the SBS-288 and SBS-1536 mutational contexts in microsatellite

stable colorectal cancers. All single base substitution (SBS) *de novo* signatures extracted using the SBS-288 (16 signatures) and SBS-1536 (14 signatures) mutational contexts, shown side by side for comparison. Equivalent signatures were not extracted in SBS-1536 format for SBS288E and SBS288K. For clarity, the signature context is retained in the signature names in this figure. The extended context for SBS-1536 signatures is omitted from the figure. Instead, the SBS-96 down-sampled version of the SBS-1536 *de novo* extracted signatures was used to display the signatures.

Supplementary Figure 13

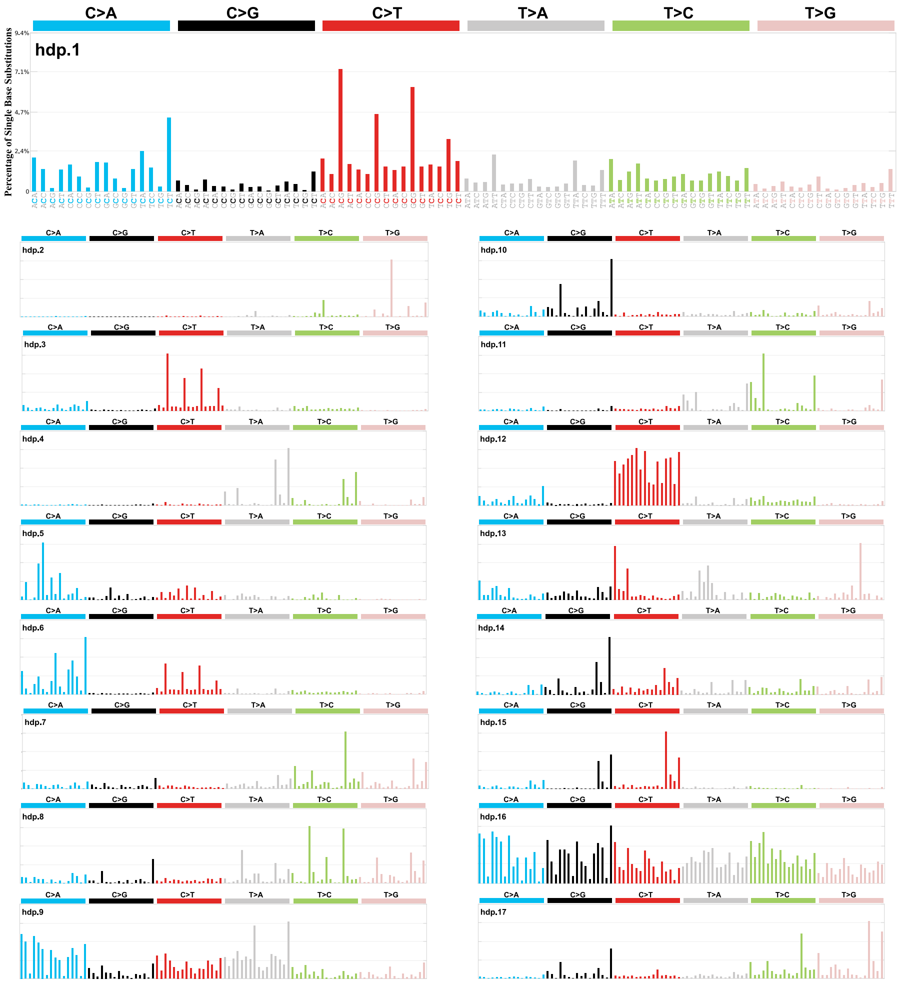

Supplementary Fig. 13. Single base substitution mutational signatures extracted by mSigHdp in microsatellite stable colorectal cancers. Seventeen single base substitution (SBS) *de novo* signatures were extracted by mSigHdp, using the SBS-96 mutational context.

Supplementary Figure 14

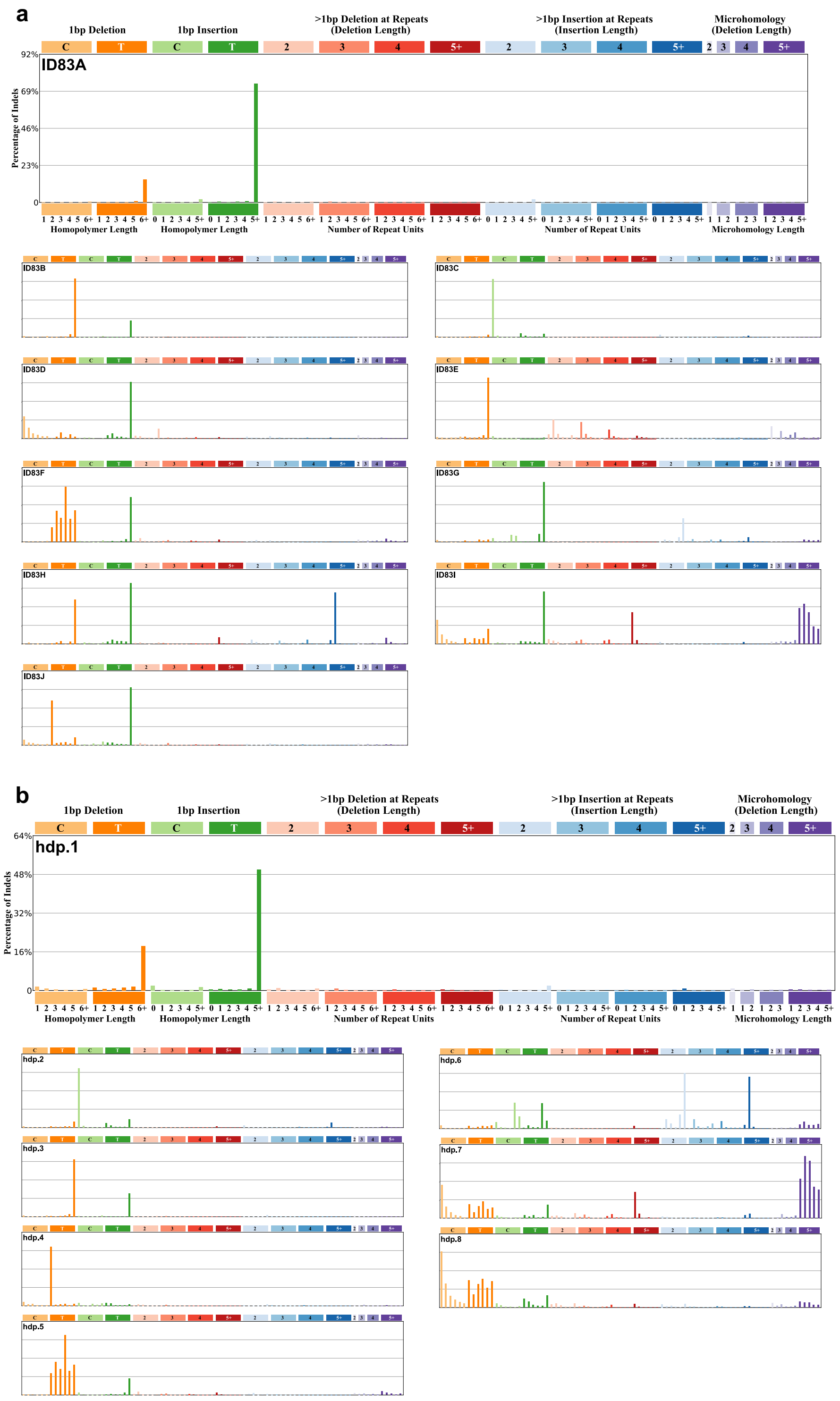

Supplementary Fig. 14. Small insertion and deletion mutational signatures extracted by SigProfilerExtractor and mSigHdp in microsatellite stable colorectal cancers. a, Ten small

insertions and deletions (ID) *de novo* signatures were extracted by SigProfilerExtractor using the ID-83 mutational context. **b**, Eight ID *de novo* signatures were extracted by mSigHdp.

Supplementary Figure 15

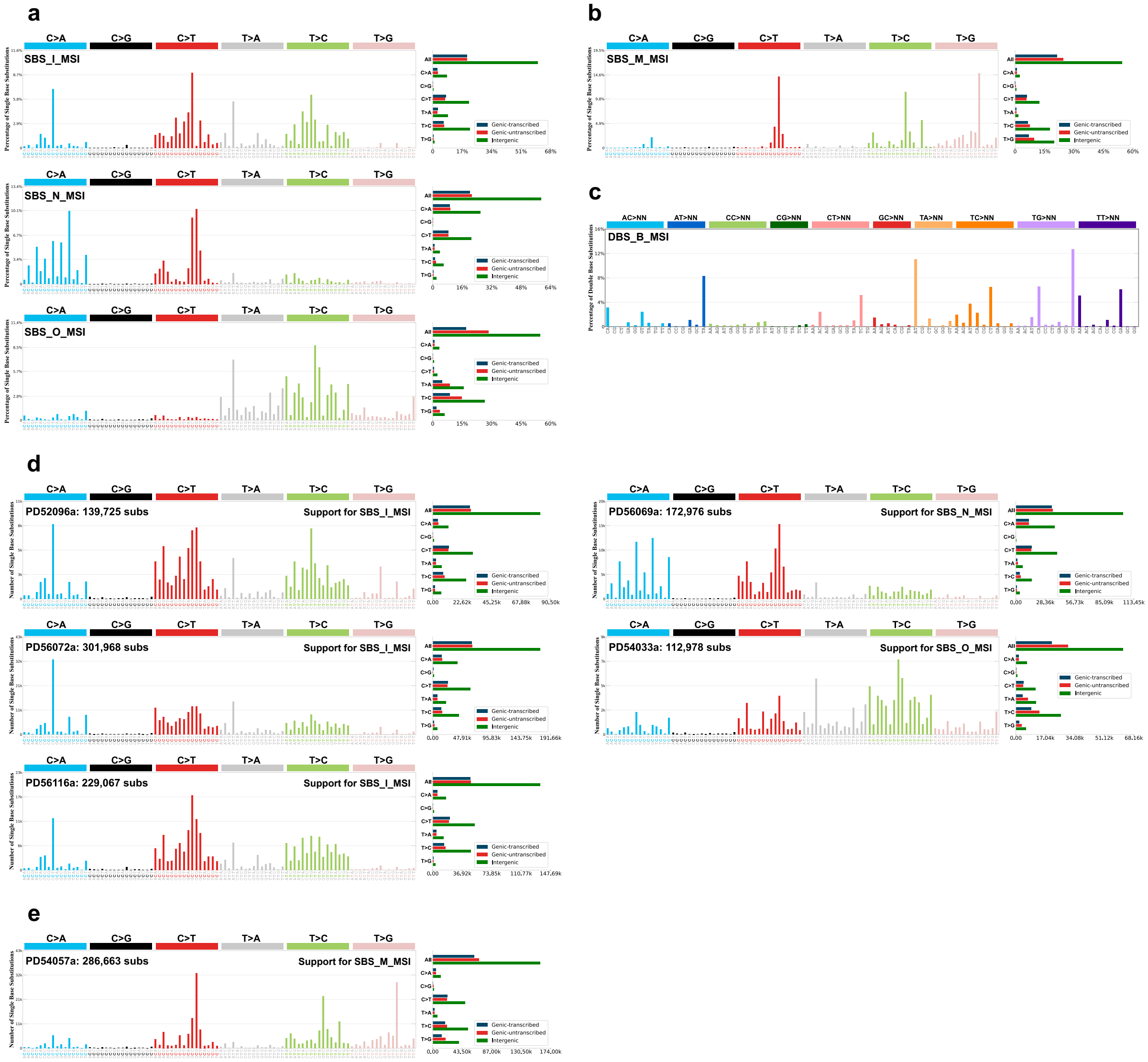

Supplementary Fig. 15. Novel mutational signatures identified in microsatellite unstable colorectal cancers. a-b, Mutational profiles of single base substitution (SBS) signatures not matching any previous COSMICv3.4 signature, including three novel signatures (**a**) and a previously reported signature (**b**). **c**, Mutational profile of a novel doublet base substitution (DBS) signature not previously reported in COSMIC. **d-e**, Exemplar mutational profiles of individual colorectal cancers supporting the three novel signatures indicated in **a** (**d**) and the previously reported signature in **b** (**e**).

Supplementary Figure 16

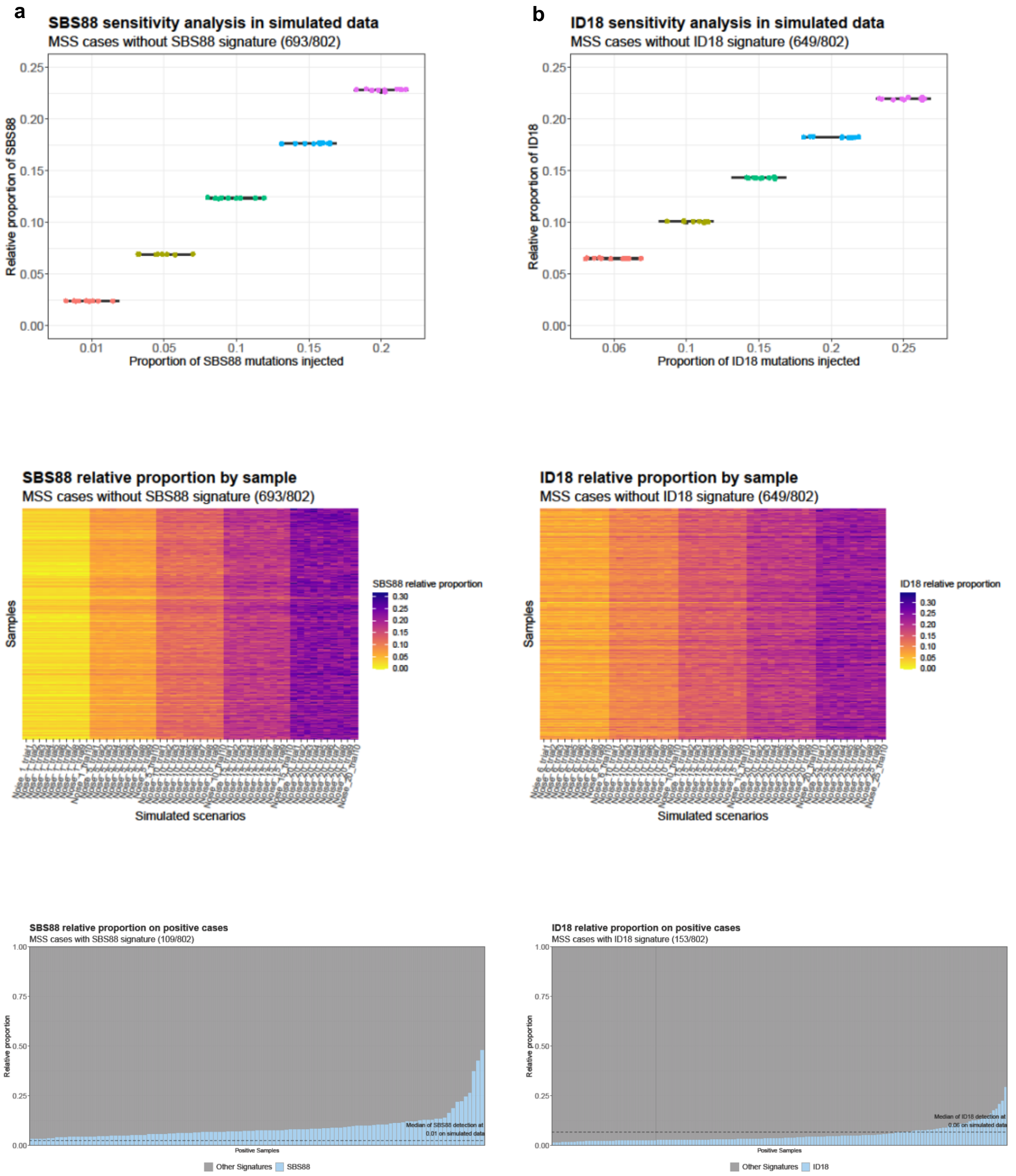

Supplementary Fig. 16. Sensitivity analysis for the detection of colibactin signatures in microsatellite stable colorectal cancers. a-b, Sensitivity analysis for SBS88 (**a**) and ID18 (**b**) detection, including average relative activity of the signature detected across all colibactin negative

samples and simulations (top), activity of the signature per sample across all colibactin negative samples and simulations (middle), and activity of the signature per sample across all colibactin positive samples compared to the median detection of the signature in the simulated data (bottom).

Supplementary Figure 17

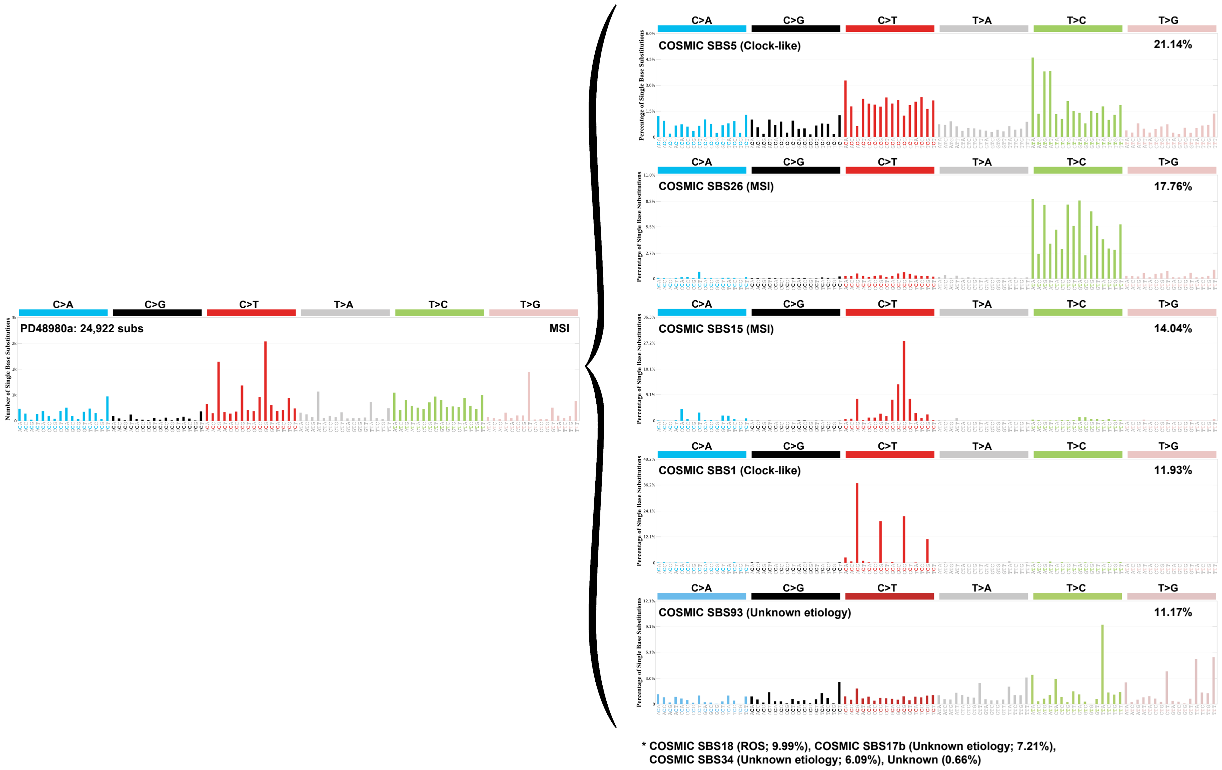

Supplementary Fig. 17. Mutational signature reconstruction of an individual microsatellite unstable tumor not validated by droplet digital PCR. Multiple mutational signatures were assigned to this case, as indicated in **Supplementary Table 37**, including microsatellite instability-associated COSMICv3.4 signature SBS15 and SBS26, suggesting the presence of microsatellite instability.

Supplementary Figure 18

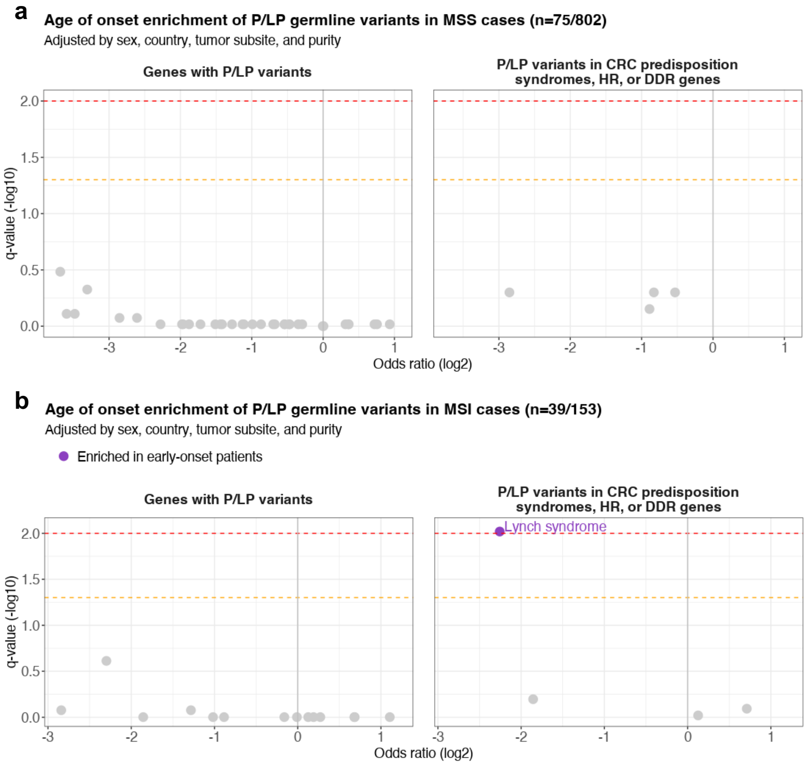

Supplementary Fig. 18. Variation of germline pathogenic variants with age of onset. a-b, Volcano plots indicating the enrichment of pathogenic or likely pathogenic germline variants in early-onset and late-onset cases in microsatellite stable (MSS; **a**) and unstable colorectal cancers (MSI; **b**). Separate analyses were performed for all individual genes (left), and for genes grouped in colorectal cancer predisposition syndromes (Lynch syndrome and Cowden syndrome), homologous recombination, or DNA damage repair-associated genes (right). Statistically significant enrichments were evaluated using multivariable logistic regression models for age of onset categorized in two subgroups (early-onset, <50 years of age; and late-onset, ≥50) and adjusted by sex, country, and tumor subsite, and tumor purity. Firth's bias-reduced logistic regressions were used for regressions presenting complete or quasi-complete separation. P-values were adjusted for multiple comparisons based on the total number of germline variants considered

and reported as q-values. Horizontal lines marking statistically significant thresholds were included at 0.05 (dashed orange line) and 0.01 q-values (dashed red line).

Supplementary Figure 19

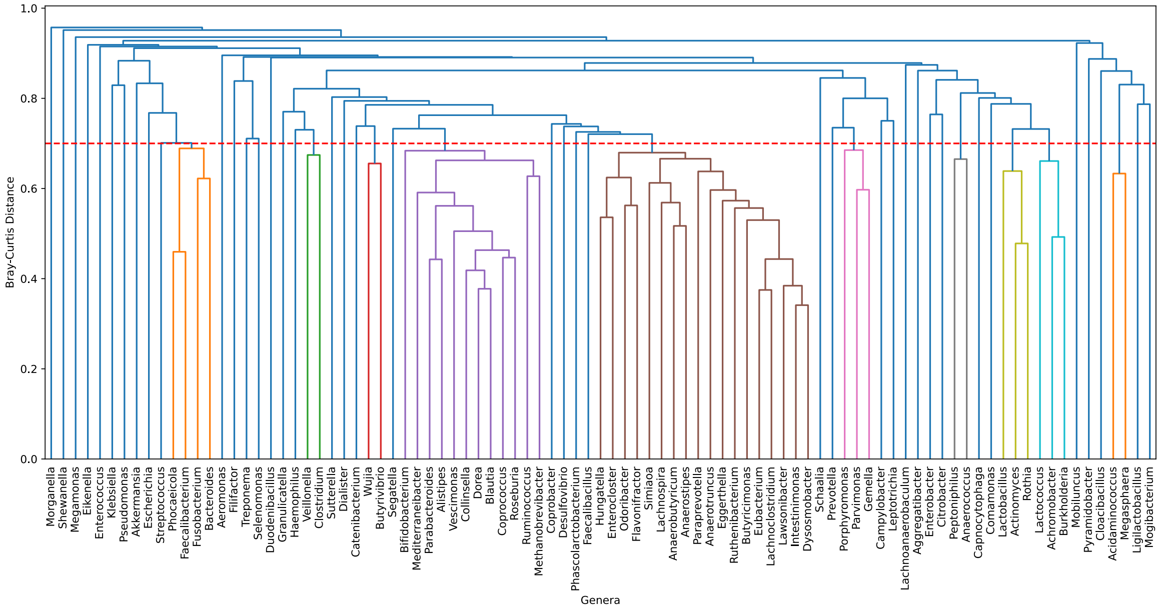

Supplementary Fig. 19. Dendrogram of the relative abundances of the considered bacterial genus based on the Bray-Curtis distance.
