# Supplementary material for: Geographic and age variations in mutational processes in colorectal cancer
Source: Nature. 2025 Apr 23;643(8070):230–40. doi: 10.1038/s41586-025-09025-8 (PMC12221974; doi:10.1038/s41586-025-09025-8)
Supplement: Supplementary file 2 — Reporting Summary [file 41586_2025_9025_MOESM2_ESM.pdf]

nature portfolio

Corresponding author(s): Ludmil B. Alexandrov

Last updated by author(s): Apr 14, 2025

Reporting Summary

Nature Portfolio wishes to improve the reproducibility of the work that we publish. This form provides structure for consistency and transparency in reporting. For further information on Nature Portfolio policies, see our Editorial Policies and the Editorial Policy Checklist.

Statistics

For all statistical analyses, confirm that the following items are present in the figure legend, table legend, main text, or Methods section.

n/a	Confirmed	
☐	☒	The exact sample size (*n*) for each experimental group/condition, given as a discrete number and unit of measurement
☐	☒	A statement on whether measurements were taken from distinct samples or whether the same sample was measured repeatedly
☐	☒	The statistical test(s) used AND whether they are one- or two-sided<br>*Only common tests should be described solely by name; describe more complex techniques in the Methods section.*
☐	☒	A description of all covariates tested
☐	☒	A description of any assumptions or corrections, such as tests of normality and adjustment for multiple comparisons
☐	☒	A full description of the statistical parameters including central tendency (e.g. means) or other basic estimates (e.g. regression coefficient) AND variation (e.g. standard deviation) or associated estimates of uncertainty (e.g. confidence intervals)
☐	☒	For null hypothesis testing, the test statistic (e.g. *F*, *t*, *r*) with confidence intervals, effect sizes, degrees of freedom and *P* value noted<br>*Give P values as exact values whenever suitable.*
☒	☐	For Bayesian analysis, information on the choice of priors and Markov chain Monte Carlo settings
☒	☐	For hierarchical and complex designs, identification of the appropriate level for tests and full reporting of outcomes
☐	☒	Estimates of effect sizes (e.g. Cohen's *d*, Pearson's *r*), indicating how they were calculated

Our web collection on statistics for biologists contains articles on many of the points above.

Software and code

Policy information about availability of computer code

Data collection

Whole genome sequencing (150bp paired end) was performed on the Illumina NovaSeq 6000 platform with target coverage of 40X for tumors and 20X for paired blood. REDCap v13.1.27 was used to collect epidemiological data.

Data analysis

Algorithms used:
Variant Calling: (https://github.com/cancerit)
BWA-Mem v0.7.16a and v0.7.17
ASCAT v4.3.3 and v4.5.0
BATTENBERG v3.5.3
cgpCaVEMan v1.11.2, v1.14.1 and v1.15.1
cgpPINDEL v2.2.5, v3.3.0 and v3.5.0
BRASS v6.1.2, v6.2.0, v6.3.0 and v6.3.4
Strelka2 v2.9.10 and Manta v1.6.0
Conpair v0.2 (https://github.com/nygenome/Conpair)
SigProfilerMatrixGenerator v1.2.0 (https://github.com/AlexandrovLab/SigProfilerMatrixGenerator)
QuantaSoft Analysis Pro v1.0.596.0525
SigProfilerExtractor v1.1.21 (https://github.com/AlexandrovLab/SigProfilerExtractor)
SigProfilerAssignment v0.0.29 (https://github.com/AlexandrovLab/SigProfilerAssignment)
MSA v2.0 (https://gitlab.com/s.senkin/MSA)
mSigHdp v2.0.1 (https://github.com/steverozen/mSigHdp)
CHORD v2.02 (https://github.com/UMCUGenetics/CHORD)
ANNOVAR v2020Jun08 (https://annovar.openbioinformatics.org/)

DPClust v2.2.8 (https://github.com/Wedge-lab/dpclust)
MutationTimeR v1.00.2 (https://github.com/gerstung-lab/MutationTimeR)
MutaGene v0.9.2.0 (https://github.com/neksa/mutagene)
Bowtie2 v2.4.2 (https://bowtie-bio.sourceforge.net/bowtie2/)
fastp v0.24.0 (https://github.com/OpenGene/fastp)
IntOGen v2023 (https://bitbucket.org/intogen/intogen-plus)
OncoKB-annotator v3.3.2 (https://github.com/oncokb/oncokb-annotator)
boostDM v2023 (https://www.intogen.org/boostdm)
logistf R package v1.26.0 (https://CRAN.R-project.org/package=logistf)
rnaturalearthdata R package v1.0.0 (https://CRAN.R-project.org/package=rnaturalearthdata)

Statistical analysis was performed in R v4.2.3
Custom code used for regression analysis and figures is available at https://github.com/AlexandrovLab/Mutographs_CRC

For manuscripts utilizing custom algorithms or software that are central to the research but not yet described in published literature, software must be made available to editors and reviewers. We strongly encourage code deposition in a community repository (e.g. GitHub). See the Nature Portfolio guidelines for submitting code & software for further information.

Data

Policy information about availability of data

All manuscripts must include a data availability statement. This statement should provide the following information, where applicable:

- Accession codes, unique identifiers, or web links for publicly available datasets
- A description of any restrictions on data availability
- For clinical datasets or third party data, please ensure that the statement adheres to our policy

Whole-genome sequencing data, somatic mutations, and patient metadata are deposited in the European Genome-phenome Archive (EGA) associated with study EGAS00001003774. All other data is provided in the accompanying Supplementary Tables.

Research involving human participants, their data, or biological material

Policy information about studies with human participants or human data. See also policy information about sex, gender (identity/presentation), and sexual orientation and race, ethnicity and racism.

Reporting on sex and gender

Sex information was self-reported and collected using epidemiological questionnaires. Overall numbers are provided in the population characteristics section of the Reporting summary. Consent was obtained for sharing individual-level data. Sex-adjusted epidemiological regressions were performed, as described in the Methods section.

Reporting on race, ethnicity, or other socially relevant groupings

The country of origin of the colorectal cancer patients was used for the regression analyses as an independent variable as described in the Methods section. This variable was not used as a proxy for any other socially constructed variables.

Population characteristics

981 cases (448 women and 533 men) diagnosed with colorectal cancer were included from the following countries: Argentina (n=53), Brazil (n=159), Canada (n=110), Colombia (n=36), Czech Republic (n=56), Iran (n=111), Japan (n=28), Poland (n=94), Russia (n=147), Serbia (n=83), and Thailand (n=104). Age at diagnosis ranged from 18 to 95 years; with a mean of 64 and a standard deviation of 12 years.

Recruitment

The International Agency for Research on Cancer (IARC/WHO) coordinated case recruitment through an international network of 17 collaborators from 11 participating countries in North America, South America, Asia, and Europe. The inclusion criteria for patients were ≥18 years of age, confirmed diagnosis of primary colorectal cancer, and no prior treatment for colorectal cancer. Informed consent was obtained for all participants. Patients were excluded if they had any condition that could interfere with their ability to provide informed consent or if there were no means of obtaining adequate tissues or associated data as per the protocol requirements. The authors are not aware of any potential self-selection bias or other biases present.

Ethics oversight

Ethical approvals were first obtained from each Local Research Ethics Committee and Federal Ethics Committee as listed below. The study was submitted and approved by the IARC/WHO Ethics Committee (IEC Project 17-10). Informed consent was obtained from all participants.
Hospital Italiano de Buenos Aires (HIBA), Buenos Aires, Argentina
A.C. Camargo Cancer Center, Sao Paulo, Brazil
Barretos Cancer Hospital, Barretos, Brazil
Hospital de Clínicas de Porto Alegre (HCPA), Porto Alegre, Rio Grande do Sul, Brazil
Ontario Tumour Bank, Ontario Institute for Cancer Research, Toronto, ON, Canada
Lunenfeld-Tanenbaum Research Institute, Sinai Health System, Toronto, ON, Canada
University Health Network, Toronto, ON, Canada
Terry Fox National Tumor Bank (Banco Nacional de Tumores Terry Fox), National Cancer Institute, Bogotá, Colombia
Charles University, Prague, Czech Republic
Tehran University of Medical Sciences, Tehran, Iran
Golestan University of Medical Sciences, Gorgan, Iran
National Cancer Center Research Institute, Chuo-ku, Japan
Nofer Institute of Occupational Medicine, Łódź, Poland
The Maria Sklodowska-Cure National Research Institute of Oncology, Warsaw, Poland
N.N. Blokhin National Medical Research Centre of Oncology, Moscow, Russia

University Clinical Centre of Serbia, Belgrade, Serbia
National Cancer Institute, Bangkok, Thailand
Chiang Mai University, Chiang Mai, Thailand
Prince of Songkla University, Hat Yai, Thailand

Note that full information on the approval of the study protocol must also be provided in the manuscript.

Field-specific reporting

Please select the one below that is the best fit for your research. If you are not sure, read the appropriate sections before making your selection.

☒ Life sciences ☐ Behavioural & social sciences ☐ Ecological, evolutionary & environmental sciences

For a reference copy of the document with all sections, see nature.com/documents/nr-reporting-summary-flat.pdf

Life sciences study design

All studies must disclose on these points even when the disclosure is negative.

Sample size	Cases were selected from prospective and retrospective studies from populations which reflect a range of colorectal cancer incidence rates. Sample sizes were limited by the number of cases available. Sample sizes are considered to be sufficient given that this is the largest cohort of whole-genome sequenced colorectal cancers ever collected across multiple countries and continents.
Data exclusions	Cases were excluded for any of the following pre-established criteria: 1) Incomplete data on core set variables (age at diagnosis and sex); 2) Failure to pass pathology review as described in the Methods; 3) If matched tumour/normal tissue did not originate from the same individual as determined by Fluidigm SNP genotyping; 4) If sequencing coverage was below 30X for tumour, or 15X for matched normal tissue; 5) Evenness of coverage criteria; 6) if contamination level was above 3% as determined by Conpair. For evenness of coverage, the median over mean coverage (MoM) score was calculated. Tumors with MoM scores outside the range of values determined by previous studies to be appropriate for whole genome sequencing (0.92 – 1.09) were excluded.
Replication	Signature extraction analysis using SigProfilerExtractor was replicated independently at both Wellcome Sanger Institute and UCSD, and signature assignment analysis using MSA was replicated at both Wellcome Sanger Institute and IARC/WHO, to ensure consistency. Regression analyses were also replicated two times independently at different institutions (UCSD and IARC/WHO). All attempts at replication were successful. No other experiments other than those mentioned here were replicated independently. Replication was not performed for additional experiments considering the standard nature of other cancer genomics analyses (such as germline and somatic variant calling, subclonal reconstruction, mutation timing, and driver gene identification), the use of the state-of-the-art tools, most of them previously used in main pan-cancer studies like PCAWG (DPClust, MutationTimeR, SigProfiler, IntOGen), and the orthogonal validation provided by the use of different methods (e.g., Strelka and cgpCaVEMan for somatic point mutation calling).
Randomization	Randomization is not relevant for this study. Cases did not undergo interventions. All cases were collected based on diagnosis of primary colorectal cancer and no prior treatment.
Blinding	Blinding is not relevant for this study. Cases were not subject to any interventions.

Reporting for specific materials, systems and methods

We require information from authors about some types of materials, experimental systems and methods used in many studies. Here, indicate whether each material, system or method listed is relevant to your study. If you are not sure if a list item applies to your research, read the appropriate section before selecting a response.

Materials & experimental systems

n/a	Involved in the study	
☒	☐	Antibodies
☒	☐	Eukaryotic cell lines
☒	☐	Palaeontology and archaeology
☒	☐	Animals and other organisms
☒	☐	Clinical data
☒	☐	Dual use research of concern
☒	☐	Plants

Methods

n/a	Involved in the study	
☒	☐	ChIP-seq
☒	☐	Flow cytometry
☒	☐	MRI-based neuroimaging

Plants

Seed stocks	N/A
Novel plant genotypes	N/A
Authentication	N/A
